# Long Run Non‐Transitivity of Win Ratio and How to Rule It Out

**DOI:** 10.1002/sim.70581

**Published:** 2026-08-19

**Authors:** Olga V. Demler, Anna Győrffy‐Kerekes

**Affiliations:** ^1^ Computer Science Department ETH Zurich Zurich Switzerland; ^2^ Brigham and Women's Hospital/Harvard Medical School Boston Massachusetts USA; ^3^ Max Planck ETH Center for Learning Systems Tübingen Germany

## Abstract

The win ratio (WR) has emerged as an increasingly used method for analyzing composite endpoints in randomized controlled trials (RCTs). Its growing popularity stems from its ability to accommodate a hierarchy of event types within a composite primary endpoint in an RCT. However, as a relatively new measure, the WR exhibits some surprising properties. Oakes and other authors have noted that for a given triplet of participants, there are realizations of event times and hierarchy of event types when “wins” form a loop. They term this property “intransitivity.” In this paper, we report and study a different phenomenon, a violation of long‐run transitivity of WRs, for example, when in the long run $\text{WR}\left(T_{A}>T_{B}\right)>1$, $\text{WR}\left(T_{B}>T_{C}\right)>1$ and paradoxically $\text{WR}\left(T_{C}>T_{A}\right)>1$, where $T_{A},T_{B},T_{C}$ denote the time‐to‐event in trial arms A, B, and C (or placebo, standard of care and novel treatment, respectively). We refer to it as Efron‐type “non‐transitivity.” Unlike Oakes‐type intransitivity, Efron‐type non‐transitivity is an inherent property of the underlying random variables. As such, it cannot be resolved by a longer observation period or larger sample size. Lumley and Gillen showed that transitivity can be guaranteed only for univariate statistics (e.g., mean, median, etc.), whereas head‐to‐head comparisons can be non‐transitive. WR and win proportion (WP) are head‐to‐head comparisons and may be non‐transitive, in which case the best treatment cannot be determined. Therefore, the non‐transitivity property challenges the utility of all head‐to‐head comparisons as efficacy measures in RCTs. In this paper, we explore the scope of this problem, focusing on identifying conditions that rule out non‐transitivity for WR, WP, and hazard ratio (HR). We apply our findings to establish transitivity in two case studies using published RCTs. This work highlights an important phenomenon that must be recognized when the WR is used as an efficacy measure in randomized controlled trials.

## 1 Introduction

The win ratio (WR) is a novel and ubiquitous measure of treatment efficacy in randomized controlled trials (RCTs) with composite primary endpoints [1, 2]. The WR summarizes pairwise comparisons of participants from two treatment arms in terms of their times to components of the primary endpoint, while accommodating the hierarchy of event types [1, 2]. Suppose, for simplicity, that the primary outcome is all‐cause death and there is no censoring. A treatment “wins” over placebo for a given pair of participants if the time‐to‐event is longer for participant i from the treatment arm (arm A) than the time‐to‐event for participant j from the placebo arm (arm B) ($t_{\mathrm{Ai}}>t_{\mathrm{Bj}}.$). The WR is the ratio of the number of wins for the treatment arm to the number of wins for the placebo arm. A WR significantly greater than 1 is interpreted as evidence of treatment efficacy over placebo. In many clinical trials, the primary endpoint is composed of several types of events. In the past, the final analysis considered only the time to the earliest event. However, the time‐to‐event for the composite primary outcome is more nuanced, and it is desirable to prioritize severe events over less severe types. The WR addresses this issue by accommodating more than one event type and their hierarchy, for example, a death can take precedence over a non‐fatal event. Thus, the WR uses more nuanced outcome information, which translates into more power [3]. When an endpoint is defined as time to the earliest event, WR is equal to the inverse of the hazard ratio (HR) when hazards are proportional in the two groups [4, 5]. These attractive properties explain why the WR has been used to report the results of several large RCTs in cardiology, neurology, and pulmonology [6, 7, 8, 9, 10]. As a novel statistic, the WR is still in development and poses several interesting questions. Exploring its properties continues to be an active, ongoing area of research [11].

### 1.1 Non‐Transitivity (Property of Distributions of Random Variables) Versus Intransitivity (Property of Realizations of Random Variables)

WR publications report a lack of transitivity of two distinct types. The first type is a property of *realizations* of random time‐to‐events. This was reported and studied by Oakes, Dong, and other authors [12, 13, 14]. The second type is a property of the *distributions* of random variables. This property was illustrated by Efron, popularized by Gardner [15, 16], and studied by Poddiakov, Lebedev, Lumley, Gillen, and others [17, 18, 19]. Let $T_{A}$ and $T_{B}$ denote the times to composite primary endpoints in two trial arms (A and B). Sometimes, for a specific triplet of participants (i, j, k), the times to components of their primary outcome form a loop, so that i wins over j, j wins over k, and k wins over i. Oakes calls this lack of transitivity “intransitive comparison” and estimates how frequently they occur when time‐to‐events are sampled from certain types of distribution functions, implying that such non‐transitive realizations can occur for transitive true WRs [13]. This lack of transitivity is a property of the specific *realization* of random variables. In contrast, the non‐transitivity that we study in this paper is defined as a property of the *distribution functions* of random variables. A famous example of this type of non‐transitivity is the set of four non‐transitive Efron dice [15, 16]. Scores on dice A, B, and C, are distributed in such a way that in the long run, die A tends to beat die B, die B tends to beat die C, and die C paradoxically tends to beat die A, or $P\left(T_{A}>T_{B}\right)>\frac{1}{2}$, $P\left(T_{B}>T_{C}\right)>\frac{1}{2}$ and $P\left(T_{C}>T_{A}\right)>\frac{1}{2}$ (here $T_{A},T_{B},T_{C}$ are random variables with categorical distribution function, the scores on dice A,B,C, respectively) (Appendix D: Example A1. Efron Dice: An Example of Non‐Transitive Discrete Random Variables).

### 1.2 WR and WP in Randomized Controlled Trials Can Be Non‐Transitive

If scores on three dice represent times to the primary endpoint of participants in an RCT with treatment arms A, B, and C, then $P\left(T_{A}>T_{B}\right)$ is called the win proportion (WP), also known as probabilistic index in clinical trial literature [20]. The Efron dice example demonstrates that WPs can be non‐transitive. Because non‐transitivity is a property of distribution functions of random variables, it is a long‐run phenomenon. In this paper, we provide examples of non‐transitive WRs and specify conditions that guarantee transitivity for WR, WP, and related statistics.

### 1.3 Literature Review and Motivation for This Paper

Early studies on non‐transitivity of head‐to‐head comparisons originated primarily in economics, particularly within rational choice theory [21]. These fields postulate that a rational choice should satisfy a set of axioms that includes transitivity. Arrow's Impossibility Theorem states that no rank‐based voting rule can satisfy the transitivity axiom when aggregating individual preferences into a collective decision [22]. This result is a formalization of the Condorcet Paradox, a cyclic preference pattern observed in voting, which had been anticipated by the 13th century scholar Ramon Lull and described by the 18th‐century philosopher Marquis de Condorcet [23, 24]. In the mid‐1960s, Bradley Efron came up with the idea of a non‐transitive set of dice. He demonstrated that one can assign scores to a set of four dice such that die A tends to beat B, B beats C, C beats D, but D beats A, forming a non‐transitive loop [15, 16]. Importantly, this is not merely a finite‐sample anomaly; such non‐transitive relationships persist in the limit, and cannot be resolved asymptotically. Non‐transitivity attracted attention in mathematics. In the 1930s and 1940s, Hugo Steinhaus, Stefan Banach, and other mathematicians discussed such paradoxes informally in the famous Scottish Café in Lviv [25]. In the late 1950s, Steinhaus and Trybuła published a summary of findings on the non‐transitive behavior of three random variables [26]. This result and the extension to n random variables were formally proved by Trybuła in a series of publications in the 1950s‐1960s [27, 28]. Bogdanov [29] and independently Komisarski [30] derived the explicit formula for the supremum of the minimum of WPs as a function of the length of the loop. More recently, Sir Timothy Gowers and collaborators explored probabilistic properties of non‐transitive dice in a blog‐based collaboration, analyzing the likelihood of forming non‐transitive loops under random score assignments [31]. Lebedev proposed conditions under which transitivity can be guaranteed, offering insight into how these paradoxes might be controlled [18]. Meanwhile, Bathke, Akritas, and Brunner developed a “casino‐style” method of statistical testing designed to overcome non‐transitivity of statistics that are based on head‐to‐head comparisons [32, 33, 34]. Using real‐world data, Alós‐Ferrer, Fehr, and Garagnani demonstrated that non‐transitive choice behavior cannot be attributed to random noise, and that econometric models explicitly incorporating non‐transitivity outperform those assuming transitivity [35]. Interestingly, in bacteriology, multiple publications show that non‐transitivity or the rock‐paper‐scissors relationship is a mechanism that promotes diversity of species. For example, in a series of wet‐lab experiments, Liao et al. showed that when a non‐transitive loop is formed by three different bacteria strains, a dominant, most toxic strain of bacteria does not always win. Instead, scenarios such as “survival of the weakest” or “survival of the enemy of the strongest” develop [36, 37]. Poddiakov and co‐authors examined the non‐transitivity phenomenon across a range of domains, including psychology, sports, and education, and proposed methods for systematically generating non‐transitive rules [38, 39]. In statistics, Brown and Hettmansperger observed that before applying the Kruskal‐Wallis test, it is advisable to test for transitivity, as non‐transitive relationships among groups can distort inference. They proposed a suitable test for this scenario [40]. Later, Lumley and Gillen extended the Arrow Impossibility Theorem and Debreu's Utility Representation Theorem to the setting of pairwise statistical comparisons in clinical research [19]. Using measure theory and topological preorders, they showed that only univariate statistics guarantee transitivity, whereas bivariate statistics—such as those used in head‐to‐head comparisons—in general, do not possess the transitivity property. Lumley and Gillen also noted that non‐transitivity might present a problem for the equipoise principle [19, 41]. Indeed, the principle of equipoise asserts that, for ethical reasons, in a RCT, a promising new treatment should only be compared to the current best available treatment (standard of care). However, if the relationship is non‐transitive, then superiority of novel treatment over standard of care does not guarantee that novel treatment is better than placebo. When including a placebo arm in a new RCT is ethically impermissible [17], we should use some alternative approach. For example, we can borrow a placebo arm from an original RCT that compared what is now the standard of care treatment to the placebo. However, this strategy introduces substantial limitations due to differences in inclusion criteria and study protocols, leading to different trial populations, violation of the principle of randomization, and resulting in confounding. To mitigate these issues, one must rely on untestable assumptions and apply techniques such as inverse probability weighting to harmonize the distribution of confounders in the borrowed trial arm. Yet even with such adjustments, unmeasured confounding remains, undermining the central benefit of randomized experiments. These limitations raise an important question: *what conditions rule out non‐transitivity of WR in the context of randomized controlled trials?* This question is the main motivation of this paper.

### 1.4 Organization of the Paper

First, after showing existence of non‐transitive loops for continuous random variables in section 2.7, we focus on sufficient conditions of transitivity of WR, WP, and HR in section 3.1, followed by necessary conditions of non‐transitivity in Section 3.2. The practical utility of these results is demonstrated through two case studies in Section 4. Further implications of these findings for clinical research are presented in Section 5.

## 2 Notation, Assumptions, and Definitions

Throughout this paper, we use uppercase letters to denote random variables and lowercase letters for their sampled realizations. For example, $T_{A}$ is a random variable and $t_{A}$ is a sample realization of $T_{A}$. We denote as A, B, and C the treatment arms in an RCT. We model times from baseline to event in treatment arms A, B, and C with random variables $T_{A},T_{B},$ and $T_{C}$, respectively. Finally, $t_{\mathrm{Ai}},t_{\mathrm{Bj}},t_{\mathrm{Ck}}$ are observed times to event for specific patients i,j,k from trial arms A,B,C, respectively. Statistics are denoted by uppercase letters, and their estimators by uppercase letters with a hat (e.g., WR and $\hat{\text{WR}}$).

### 2.1 Assumptions

In this paper, we will use the following assumptions:

Assumption 1 $T_{A},T_{B},T_{C}$ are independent.

Assumption 2 There is no censoring (we assume this for simplicity).

Assumption 3 $\;P(T_{A}=T_{B})=P(T_{B}=T_{C})=P(T_{C}=T_{A})=0.$

For results involving the HR, we introduce one additional assumption:

Assumption 4 **(Proportional Hazards (PH) assumption)** The hazard ratio is constant over time.

For simplicity, we assume that the outcomes considered in this paper are simple (i.e., non‐hierarchical). The rationale for these assumptions is presented in Appendix A.

### 2.2 Win Proportion (WP)

The WP is defined as, ${\text{WP}}_{\mathrm{AB}}=P\left(T_{A}>T_{B}\right)$. To estimate WP, we form all possible pairs of participants (i,j), one from arm A with another from arm B. The WP is estimated as, ${\hat{\text{WP}}}_{\mathrm{AB}}=\frac{1}{N}\sum I\left[t_{\mathrm{Ai}}>t_{\mathrm{Bj}}\right]$, where N is the number of all possible pairs and I[·] is an indicator function, see Bebu, Lachin, Wang, Pocock for its asymptotic properties [14, 42]. WP varies from 0 to 1, with values above 0.5 supporting the efficacy of treatment A over treatment B. To test $H_{0}:{\text{WP}}_{\mathrm{AB}}>1/2$, we can take advantage of the equivalence of WP and Mann–Whitney statistics and use the asymptotic property of the latter. WP is also known as the probabilistic index [43, 44].

### 2.3 Win Ratio (WR)

The WR is defined as $WR_{\mathrm{AB}}=\frac{P\left(T_{A}>T_{B}\right)}{P\left(T_{A}<T_{B}\right)}$, and can be estimated as ${\hat{\text{WR}}}_{\mathrm{AB}}=\frac{\sum I\left[t_{\mathrm{Ai}}>t_{\mathrm{Bi}}\right]}{\sum I\left[t_{\mathrm{Ai}}<t_{\mathrm{Bj}}\right]}$, where I[·] is an indicator function [1, 2]. Therefore, $WR_{\mathrm{AB}}>1$ means that treatment A is beneficial over treatment B. Therefore, if $P\left(T_{A}=T_{B}\right)=0$ (Assumption 3 in Section 2.1), then $\text{WR}=\frac{\text{WP}}{1-\text{WP}}$. If we have no censoring either, then the estimates also have the same relationship.

### 2.4 Win Odds (WO)

The WO is defined as in Dong et al. [43] $WO_{\mathrm{AB}}=\frac{P\left(T_{A}>T_{B}\right)+\frac{1}{2}P\left(T_{A}=T_{B}\right)}{P\left(T_{A}<T_{B}\right)+\frac{1}{2}P\left(T_{A}=T_{B}\right)}$ and ${\hat{\mathrm{WO}}}_{\mathrm{AB}}=\frac{\sum I\left[t_{\mathrm{Ai}}>t_{\mathrm{Bj}}\right]+\frac{1}{2}\sum I\left[t_{\mathrm{Ai}}=t_{\mathrm{Bj}}\right]}{\sum I\left[t_{\mathrm{Ai}}<t_{\mathrm{Bj}}\right]+\frac{1}{2}\sum I\left[t_{\mathrm{Ai}}=t_{\mathrm{Bj}}\right]}$. The WR and the WO are approximately equivalent as measures of treatment effect when the number of ties and censoring is small [20]. In this paper, we assume no censoring (Assumption 2, Section 2.1) and no overlaps in outcomes (Assumption 3, Section 2.1). WR and WO are complementary measures of treatment effect (Dong et al. [20]). However, since $WR_{\mathrm{AB}}={\mathrm{WO}}_{\mathrm{AB}}$ under our assumptions, WO will not be further discussed in this paper.

### 2.5 Hazard Ratio (HR)

The HR is commonly used as a primary efficacy measure in RCTs, usually in the Cox proportional hazards model [45]. Oakes, Dong, and Gillen demonstrated that, under the proportional hazards assumption, $\mathrm{HR}=\frac{1}{\text{WR}}$ [5, 12, 46]. We also included a proof of this in Appendix B. This relationship holds only when the HR is estimated from a univariable Cox regression model that includes treatment arm as the sole predictor, without adjustment for other covariates such as age or gender.

Under Assumptions 1, 2, 3 (Section 2.1), WP and WR are monotonic transformations of one another. Additionally, under Assumptions 1, 2, 3, 4 (Section 2.1), HR is a monotonic transformation of WR and WP. We summarize the relationships between WP, WR, and HR in Table 1. We derive relationships from Table 1 in Appendix B.

**TABLE 1:** Relationships between win proportion (WP), win ratio (WR), and hazard ratio (HR). As everywhere in this paper, we assume Assumptions 1, 2, 3 (Section 2.1).

	WP	WR	HR
WP	—	$\frac{\text{WR}}{1+\text{WR}}$	$\frac{1}{1+\mathrm{HR}}$ *
WR	$\frac{\text{WP}}{1-\text{WP}}$	—	$\frac{1}{\mathrm{HR}}$ *
HR	$\frac{1-\text{WP}}{\text{WP}}$	$\frac{1}{\text{WR}}$	—

* Relies on the assumption that all events are observed and the proportional hazards assumption holds.

### 2.6 Transitivity and Non‐Transitivity

We define transitivity and non‐transitivity as the long‐run behavior of random variables, following the example of Efron's dice [15]. In the context of this paper, the relevant random variables represent times‐to‐events.

Consider three times‐to‐events $T_{A},T_{B}$ and $T_{C},$ and assume ${\text{WP}}_{\mathrm{AB}}=P\left(T_{A}>T_{B}\right)>\frac{1}{2}$ and ${\text{WP}}_{\mathrm{BC}}=P\left(T_{B}>T_{C}\right)>\frac{1}{2}$. We call $T_{A},T_{B}$ and $T_{C}$ transitive with respect to WP if ${\text{WP}}_{\mathrm{AC}}=P\left(T_{A}>T_{C}\right)>\frac{1}{2}$ (equivalently ${\text{WP}}_{\mathrm{CA}}<\frac{1}{2}$) and non‐transitive if ${\text{WP}}_{\mathrm{AC}}\leq \frac{1}{2}$ (equivalently ${\text{WP}}_{\mathrm{CA}}\geq \frac{1}{2}$).

Transitivity and non‐transitivity of WR and HR are defined analogously. Due to the monotonic relationships between WP and WR, establishing non‐transitivity (or transitivity) in one of them implies non‐transitivity (or transitivity) in the other. Similarly, under the PH Assumption (Section 2.1, Assumption 4), non‐transitivity (or transitivity) of HR implies non‐transitivity (or transitivity) in WR and WP and vice versa. (see Appendix C for the proof).

### 2.7 Existence of Non‐Transitive Loops Formed by WP of Three Independent Random Variables

Statement 1 *WPs can form non‐transitive loops of length* 3.

We prove by example the existence of non‐transitive loops with respect to WP. We construct two explicit examples: one for discrete random variables and one for continuous random variables.

Example 1 Non‐transitive loop formed by three discrete random variables [27].

If $T_{A},T_{B}$ and $T_{C}$ are discrete random variables that take values in the set {1,2,3,4,5} with probabilities defined in Table 2, then $WP_{\mathrm{AB}}>1/2,WP_{\mathrm{BC}}>1/2,$ and ${\text{WP}}_{\mathrm{CA}}>1/2$. Indeed $P\left(T_{A}>T_{B}\right)=0.4+0.6\times 0.3=0.58,P\left(T_{B}>T_{C}\right)=0.7,$ and $P\left(T_{C}>T_{A}\right)=0.6$.

Example 2 Non‐transitive loop formed by three continuous random variables.

We use the following relationship. $P\left(T_{A}>T_{B}\right)=\int_{-\infty }^{\infty }F\left(t_{B}\right)\mathrm{dG}\left(t_{B}\right)=\int_{-\infty }^{\infty }F\left(t_{B}\right)g\left(t_{B}\right)dt_{B}=\int_{-\infty }^{\infty }\left(1-G\left(t_{A}\right)\right)\mathrm{dF}\left(t_{A}\right)=\int_{-\infty }^{\infty }\left(1-G\left(t_{A}\right)\right)f\left(t_{A}\right)dt_{A}$, where $T_{A}∼F\left(\cdot \right),T_{B}∼G\left(\cdot \right)$.

**FIGURE 1:**
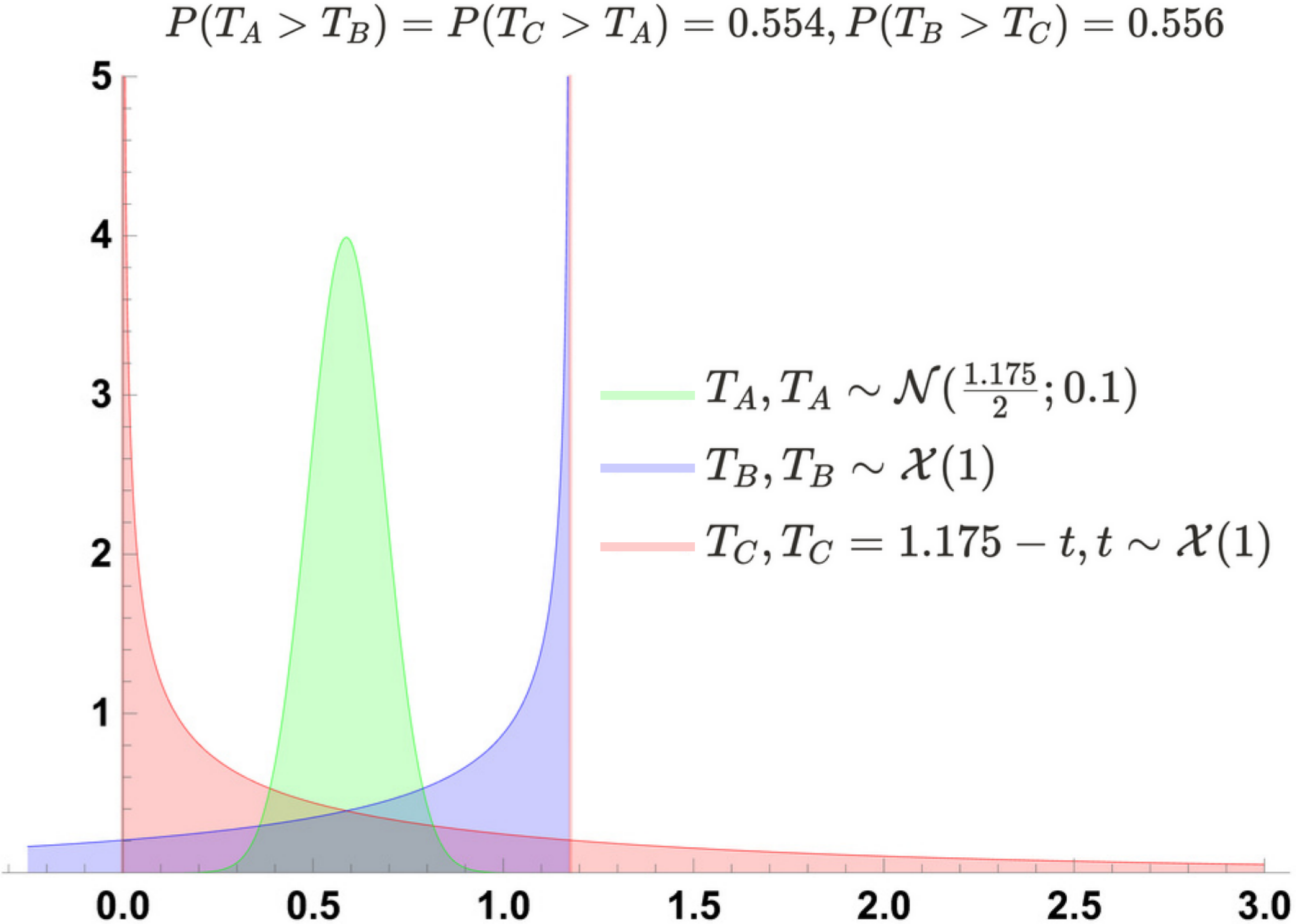
Three non‐transitive continuous random variables.

If $T_{A}∼N\left(\mu =\frac{1.175}{2},\sigma =0.1\right)$, $T_{B}∼\chi_{1}\left(\cdot \right)$ and $T_{C}∼1.175-t,t∼\chi_{1}\left(\cdot \right)$, where $\chi_{1}\left(\cdot \right)$ is Chi‐squared distribution with 1 degree of freedom. Then, ${\text{WP}}_{\mathrm{AB}}=0.554,$ ${\text{WP}}_{\mathrm{BC}}=0.556,{\text{WP}}_{\mathrm{CA}}=0.554,$ and therefore, non‐transitivity is established. The plot of probability density functions for Example 2 is in Figure 1.

**TABLE 2:** Three discrete non‐transitive random variables [27].

	1	2	3	4	5
$T_{A}$	0.0	0.6	0.0	0.0	0.4
$T_{B}$	0.3	0.0	0.0	0.7	0.0
$T_{C}$	0.0	0.0	1.0	0.0	0.0

Examples 1 and 2 prove the existence of three random variables that form non‐transitive loops with respect to WP. Using Appendix C, we can extend this result to WR statistics.

**Note 1**. The discrete distributions in Example 1 are a special case of distribution functions from the works of Steinhaus and Trybuła on non‐transitivity [26, 27].

**Note 2**. More examples of non‐transitive loops are presented in the Appendix D: Example A1. Efron Dice: An Example of Non‐Transitive Discrete Random Variables, Example A2, Example A3, Example A4, [15].

## 3 Methods

Having established that WP and WR can form non‐transitive loops, we now investigate the conditions under which non‐transitivity can be ruled out. We show that certain *distribution functions* can guarantee transitivity (Section 3.1.1). We also show that certain *magnitudes* of WPs can guarantee transitivity (Section 3.1.3). In Section 3.1.5, we discuss the transitivity of HR. Finally, in Section 3.2, we present the necessary conditions for non‐transitivity. As mentioned before, the results of Sections 3.1 and 3.2 apply to WP and WR under Assumptions 1, 2, 3 (Section 2.1), with the exception of Section 3.1.5, where we also need Assumption 4 (Section 2.1). Some of the lemmas below were proved elsewhere, as referenced; the remaining proofs are presented in the Appendix.

### 3.1 Sufficient Conditions

#### 3.1.1 Sufficient Conditions on Distribution Functions that Guarantee Transitivity of WPs of Three Random Variables

In this section, we formulate conditions on distribution functions that guarantee transitivity of the probability that one variable is greater than the other one (=WP). Proofs are presented in Appendix E. In this section, we step outside the framework of RCTs and formulate Lemmas 1, [Link], 2, [Link], 3, [Link], 4, [Link], 5, [Link], 6 for independent random variables. If we also want to apply these Lemmas to WR, we need to assume Assumptions 1, 2, 3 from Section 2.1. We also discuss how to put these conditions in the context of RCTs.

Lemma 1 *WPs generated by independent binary 0‐1 random variables are always transitive*.

In Appendix E: Lemma 1. Three Binary Random Variables Are Always Transitive.

Lemma 2 *WPs generated by independent normally distributed random variables are always transitive*.

In Appendix E: Lemma 2. Three Normally Distributed Random Variables Are Always Transitive.

Lemma 3 *WPs generated by independent log‐normally distributed random variables are always transitive*.

In Appendix E: Lemma 3. Three Log‐Normally Distributed Random Variables Are Always Transitive.

Lemma 4 *WPs generated by independent continuous random variables with symmetric probability density functions (PDFs) around their* mean (=median) *are always transitive*.

In Appendix E: Lemma 4. Three Continuous Random Variables With Symmetric Pdfs Are Always Transitive.

Normal, Uniform, Student's *t*, Logistic, and Cauchy distributions have symmetric density functions. Random variables with symmetric distribution functions can still form non‐transitive loops, but only when combined with random variables with asymmetric PDFs, such as Chi‐squared, exponential, beta, etc. Illustrative examples can be found in Example 2 within the proof of Statement 1, as well as in Appendix D: Example A2, Example A3, Example A4.

Lemma 5 *WPs generated by independent random variables with the same PDFs up to an additive shift are always transitive*[27].

In Trybuła, 1961, Theorem 2 [27].

Lemma 6 *WPs generated by three independent random variables with polynomial of degree* 3 *and lower cumulative distribution functions defined on the interval* [0, 1] *are always transitive* [18].

In Lebedev, 2019, Proposition 2 [18].

We can further extend the family of distribution functions that are always transitive by noting that a monotonic transformation of three or more transitive random variables also yields transitive random variables.

#### 3.1.2 Practical Implications of Lemmas 1, [Link], 2, [Link], 3, [Link], 4, [Link], 5, [Link], 6

Sufficient conditions for transitivity, presented in Section 3.1.1, limit the set of distribution functions that can form non‐transitive loops.

A discussion on extending these results to accommodate censoring and tied event times is provided in Appendix I. We are currently working on further extensions of these findings.

*Applications to RCTs with surrogate endpoints*. Lemmas 1, [Link], 2, [Link], 3, [Link], 4, [Link], 5, [Link], 6 may be useful when comparing the continuous surrogate endpoints. Although many biomarkers seem to follow bell‐shaped distribution functions [47], it is not clear whether the normality assumption or any of the conditions specified in sufficiency Lemmas 1, [Link], 2, [Link], 3, [Link], 4, [Link], 5, [Link], 6 may hold when comparing changes of the biomarker in response to treatment.

*Applications to loops of length* > *3*. Lemmas 1, [Link], 2, [Link], 3, [Link], 4, [Link], 5 hold for loops of any length. Lemma 6 is restricted to loops of length 3.

#### 3.1.3 Sufficient Conditions on the Magnitude of WPs and WRs That Guarantee Transitivity of Three Variables

The next question we address is whether the magnitude of WR or WP can be high enough to guarantee transitivity (Section 3.1.3). In this section and the rest of the paper, we assume that Assumptions 1, 2, 3 from Section 2.1 are true.

Lemma 7 *For any three random variables* $T_{A},T_{B}$ *and* $T_{C}$ *such that Assumptions* 1, 2, 3, *Section* 2.1 *are satisfied*,

*(1) if* ${\mathrm{WP}}_{\mathrm{BC}}\geq \frac{1}{2{\mathrm{WP}}_{\mathrm{AB}}}$, *then* $T_{A},T_{B}$ *and* $T_{C}$ *are transitive that is* ${\mathrm{WP}}_{\mathrm{AC}}\geq \frac{1}{2}$.

*(2) if* ${\mathrm{WR}}_{\mathrm{BC}}\geq \frac{1+{\mathrm{WR}}_{\mathrm{AB}}}{{\mathrm{WR}}_{\mathrm{AB}}-1},$ *then* $T_{A},T_{B}$ *and* $T_{C}$ *are transitive that is* ${\mathrm{WR}}_{\mathrm{AC}}\geq 1.$

Proof of Lemma 7 is provided in Appendix E: Lemma 7 [27]. (1) follows directly from Corollary 1.4 of Theorem 1 in Trybuła, 1961 [27]. (2) follows from (1) and the monotonic relationship between WP and WR (Appendices B and C). Since Trybuła did not include proof of Corollary 1.4, for completeness, we present it in Appendix H: Corollary 1.4.

#### 3.1.4 Practical Implications of Lemma 7

**FIGURE 2:**
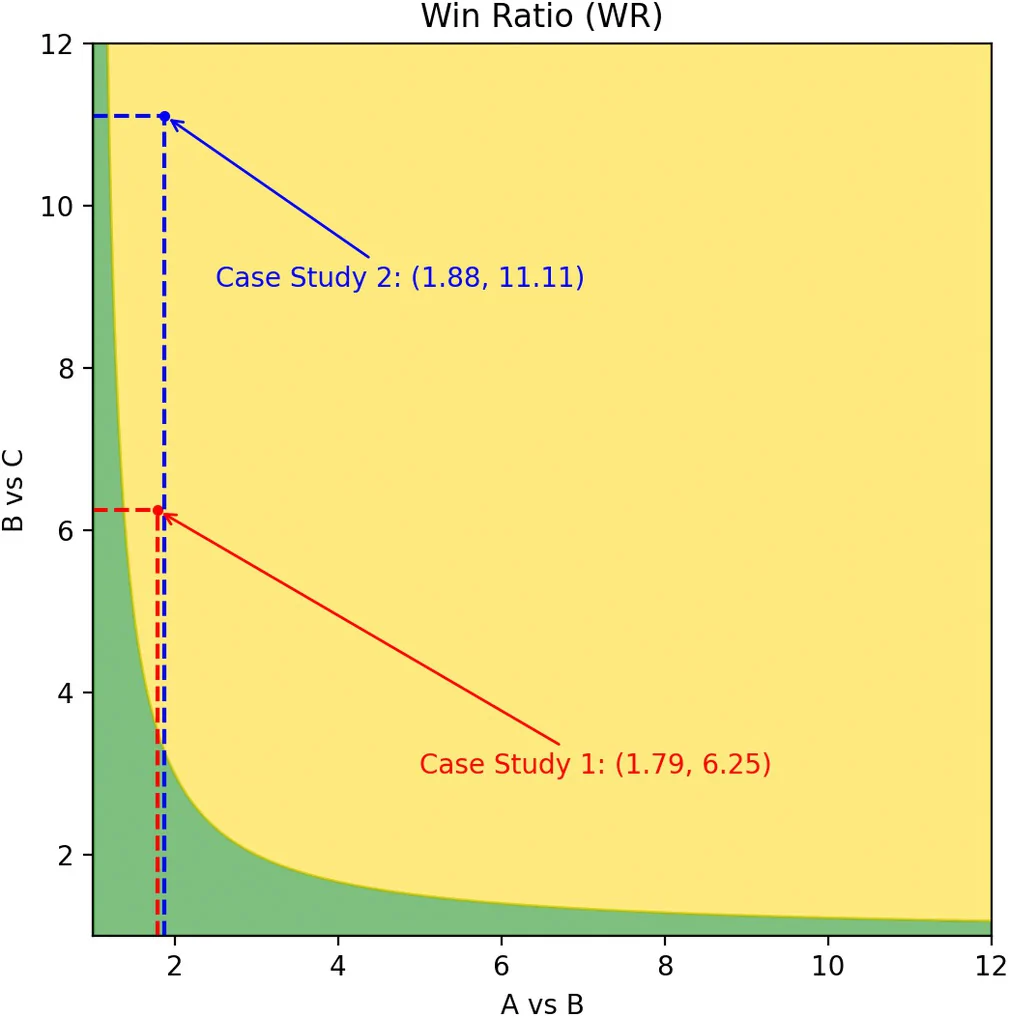
Transitive region (yellow). Both transitive and non‐transitive comparisons are possible in the green region. *Case Study 1:* The treatments are (A) **Dabigatran**, (B) **Warfarin**, and (C) **Standard of care in the 1980s**. Then ${\hat{\text{WR}}}_{\mathrm{AB}}={\hat{\text{WR}}}_{d\;\mathrm{vs}.\;w\;|\;\text{women}}=1.79$ and ${\hat{\text{WR}}}_{\mathrm{BC}}={\hat{\text{WR}}}_{w\;\mathrm{vs}.\;\mathrm{soc}\;|\;\text{women}}=6.25$. *Case Study 2:* The treatments are (A) **TAVR**, (B) **SAVR**, and (C) **Non‐surgical (NS)**. Then ${\hat{\text{WR}}}_{\mathrm{AB}}={\hat{\text{WR}}}_{\text{TAVR}\;\mathrm{vs}.\;\text{SAVR}}=1.88$ and ${\hat{\text{WR}}}_{\mathrm{BC}}={\hat{\text{WR}}}_{\text{SAVR}\;\mathrm{vs}.\;\mathrm{NS}}=11.11$. WR estimates in case studies 1 and 2 are in the yellow region, and therefore by Lemma 7 we establish transitivity.

Lemma 7 implies that when the magnitude of two comparisons is large enough, then transitivity is guaranteed. In Figure 2, we plot two WRs and the boundary that separates the yellow region, where transitivity is guaranteed by Lemma 7, from the green region, where transitivity/non‐transitivity status is moot. Lemma 7 implies that if the original comparison of standard of care to placebo and novel treatment to standard of care falls in the yellow region plotted in Figure 2, then the relationship is transitive, and therefore, novel treatment is guaranteed to be better than placebo. Indeed, when comparing novel treatment (NT), standard of care (SOC), and placebo (P) if ${\text{WR}}_{\mathrm{NT}\;\mathrm{vs}.\;\mathrm{SOC}}\geq \frac{1+{\text{WR}}_{\text{SOC vs. P}}}{{\text{WR}}_{{\text{SOC vs. P}}^{-1}}}$, then ${\text{WR}}_{\mathrm{NT}\;\mathrm{vs}.\;P}\geq 1$ by Lemma 7, and therefore, direct comparison of novel treatment with placebo is not needed. We can call $\frac{1+{\text{WR}}_{\text{SOC vs. P}}}{{\text{WR}}_{{\text{SOC vs. P}}^{-1}}}$ as “a threshold of transitivity.” This threshold can be calculated from the original trial by comparing SOC to P. Results of subsequent trials testing the efficacy of NT versus SOC can be compared to this threshold. We illustrate how Lemma 7 can be used to guarantee transitivity in Case Studies 1 and 2.

Note that the assumptions used in Lemma 7 are milder than the assumptions needed for direct comparison across two different trials. Indeed, Assumptions 1, 2, 3 do not require that the randomization principle was followed.

#### 3.1.5 Sufficient Conditions for the Hazard Ratio

Statement 2 *If proportionality of hazards (*Assumption 4, Section 2.1 *) holds for all comparisons, then HRs are transitive*.

In Appendix E: Statement 2.

Statement 2 means that proportionality of hazards implies transitivity of the HR as well as WR and WP. However, if the PH assumption does not hold in all trials, we cannot use Statement 2 to establish transitivity.

### 3.2 Necessary Conditions for Non‐Transitive Loops

Sufficient conditions for transitivity presented above restrict the set of distribution functions and the strength of WR and WP that can form non‐transitive loops. To further investigate the properties of non‐transitive loops of length 3, we study their necessary conditions. Proofs are presented in the Appendix F.

#### 3.2.1 Necessary Conditions Lemma 8

Lemma 8 *Suppose three random variables* $T_{A},T_{B}$ *and* $T_{C}$ *satisfy Assumptions* *1*, *2*, *3* *(Section* *2.1* *). If they form a non‐transitive loop, then the following equivalent statements hold:*

*(1) At least one of their WPs is less than or equal to* $\frac{\sqrt{5}-1}{2}\approx 0.618$ [29, 30].

*(2) At least one of their WRs is less than or equal to* $\frac{1+\sqrt{5}}{2}\approx 1.618$.

Proof relies on solution 10.5 from Bogdanov [29] or, equivalently, Theorem 1 from Komisarski [30] when the length of the loop is 3 (Appendix F: Lemma 8).

#### 3.2.2 Practical Implications of Lemma 8

Lemma 8 implies that if $WR_{\mathrm{SOC}\;\mathrm{vs}.P}\leq 1.618$ or $WR_{\mathrm{NT}\;\mathrm{vs}.\;\mathrm{SOC}}\leq 1.618$, then the necessary condition for non‐transitivity is satisfied for loops of length 3. We can still rule out non‐transitivity using Lemmas 1, [Link], 2, [Link], 3, [Link], 4, [Link], 5, [Link], 6, [Link], 8 or Statement 2.

Note that $\frac{\sqrt{5}-1}{2}$, is $\frac{1+\sqrt{5}}{2}-1,$ where $\frac{1+\sqrt{5}}{2}$ is the Golden Ratio.

Lemma 9 is an extension of Lemma 8 to loops of any length.

#### 3.2.3 Necessary Conditions Lemma 9

Lemma 9 *For all* n∈ℕ *and* n≥3, *we have* $\underset{S\in S_{n}^{o}}{\sup}\;\min_{\mathrm{WP}\in S}\mathrm{WP}=1-\frac{1}{4\cos^{2}\frac{\pi }{n+2}}$, *where* $S_{n}^{o}$ *is the space of non‐transitive sets of WPs formed by* n≥3 *independent random variables* [29, 30].

We use solution 10.5 from Bogdanov [29] or Theorem 1 by Komisarski [30].

**Note**. As stated by Bogdanov and Komisarski, for all n, this boundary is sharp.

#### 3.2.4 Practical Implications of Lemma 9

Lemma 9 implies that the longer the loop is, the higher the probabilities can be. In other words, the upper boundary of the minimal probability in a loop goes up with the length of the loop. For n=3, $\underset{S\in S_{3}^{o}}{\sup}\;\min_{\mathrm{WP}\in S}\mathrm{WP}=1-\frac{1}{4\cos^{2}\frac{\pi }{3+2}}=\frac{\sqrt{5}-1}{2}$. As n goes to infinity, this supremum is $\frac{3}{4}$ in the limit, and therefore, a sufficiently long non‐transitive loop with all WPs above 0.74 is possible. It follows that shorter non‐transitive loops must include weaker WPs, and a non‐transitive loop of any finite length cannot have all WP≥0.75.

For applications, it is important to know if there is a WP high enough that it rules out non‐transitivity. In other words, it is important to find $\underset{S\in S_{n}^{o}}{\sup}\;\max_{\mathrm{WP}\in S}\mathrm{WP}$, to see whether we can use it to guarantee that there can be no instances of non‐transitivity in a dataset. Unfortunately, the answer is “no,” at least for non‐transitive loops of length 3.

#### 3.2.5 Necessary Conditions Lemma 10

***Lemma*** 10. *Under Assumptions* 1, 2, 3 *of Section* 2.1,

$$\underset{S\in S_{n}^{o}}{\sup}\;\max_{\text{WP}\in S}\text{WP}=1$$

In Appendix F: Lemma 10.

#### 3.2.6 Practical Implications of Lemma 10

Lemma 10 implies that even if there is a novel treatment that has almost perfect WP when compared to SOC, it still can be part of a non‐transitive loop. In other words, $WP_{\mathrm{NT}\;\mathrm{vs.}\;\mathrm{SOC}}\geq 0.99$ does not guarantee transitivity. However, we can use Lemma 7, and if ${\text{WP}}_{\mathrm{SOC}\;\mathrm{vs}.\;P}\cdot {\text{WP}}_{\mathrm{NT}\;\mathrm{vs}.\;\mathrm{SOC}}\geq \frac{1}{2},$ then transitivity is guaranteed and direct comparison of novel treatment with placebo is not required. If ${\text{WP}}_{\mathrm{NT}\;\mathrm{vs}.\;\mathrm{SOC}}=0.99$, transitivity is guaranteed if ${\text{WP}}_{\mathrm{SOC}\;\mathrm{vs}.\;P}\geq \frac{0.5}{0.99}\approx 0.505$, which is quite low. We will use this approach to establish the transitivity of novel treatment, standard of care, and placebo in two case studies presented in Section 4.

### 3.3 Space $S_{3}$: Space of Win Proportions Generated by Three Independent Random Variables Under Assumptions 1, 2, 3, Section 2.1

After listing sufficient conditions of transitivity and necessary conditions of non‐transitivity, here we characterize the space of all possible triplets of WPs under Assumptions 1, 2, 3 of Section 2.1. Let us denote as $S_{3}$ the space of 3 WPs formed by $T_{A},T_{B}$ and $T_{C}:$ $S_{3}=\left\{{\text{WP}}_{\mathrm{AB}},{\text{WP}}_{\mathrm{BC}},{\text{WP}}_{\mathrm{CA}}\right\}$, and as $S_{3}^{o}$ a non‐transitive subspace of $S_{3}$.

WP can take values between 0 and 1. Hence, $S_{3}$ is a subspace of [0;1]×[0;1]×[0;1]. Non‐transitive loops are a subset of $S_{3}$. It follows from Lemma 9 that a triplet of WPs (0.8,0.8,0.8) cannot be formed by three random variables that satisfy Assumptions 1, 2, 3 of Section 2.1. This implies that S_3_ does not fill up the whole unit cube. Therefore, we could ask: if $S_{3}$ is not the whole unit cube, then how can we describe it? Lemma 11 answers this question.

Lemma 11 *Under Assumptions* 1 *–* 3 (Section 2.1) $S_{3}$ *and* $S_{n}^{o}$ *can be written as follows:*

$$\begin{array}{cc} & S_{3}=\left(\underset{A,B,C\;\text{cycl}}{⋃}\left\{\text{WP}:WP_{\mathrm{AB}}\cdot WP_{\mathrm{CA}}<1-WP_{\mathrm{BC}}\right\}\right)\\ & \;⋂\left(\underset{A,B,C\;\text{cycl}}{⋃}\left\{\text{WP}:\left(1-WP_{\mathrm{AB}}\right)\cdot \left(1-WP_{\mathrm{CA}}\right)<WP_{\mathrm{BC}}\right\}\right) \end{array}$$

$$S_{3}^{o}=S_{3}⋂\left\{\text{WP}:{\text{WP}}_{\mathrm{AB}}\geq \frac{1}{2},{\text{WP}}_{\mathrm{BC}}\geq \frac{1}{2},{\text{WP}}_{\mathrm{CA}}\geq \frac{1}{2}\right\}$$

In Appendix G: Lemma 11.

**FIGURE 3:**
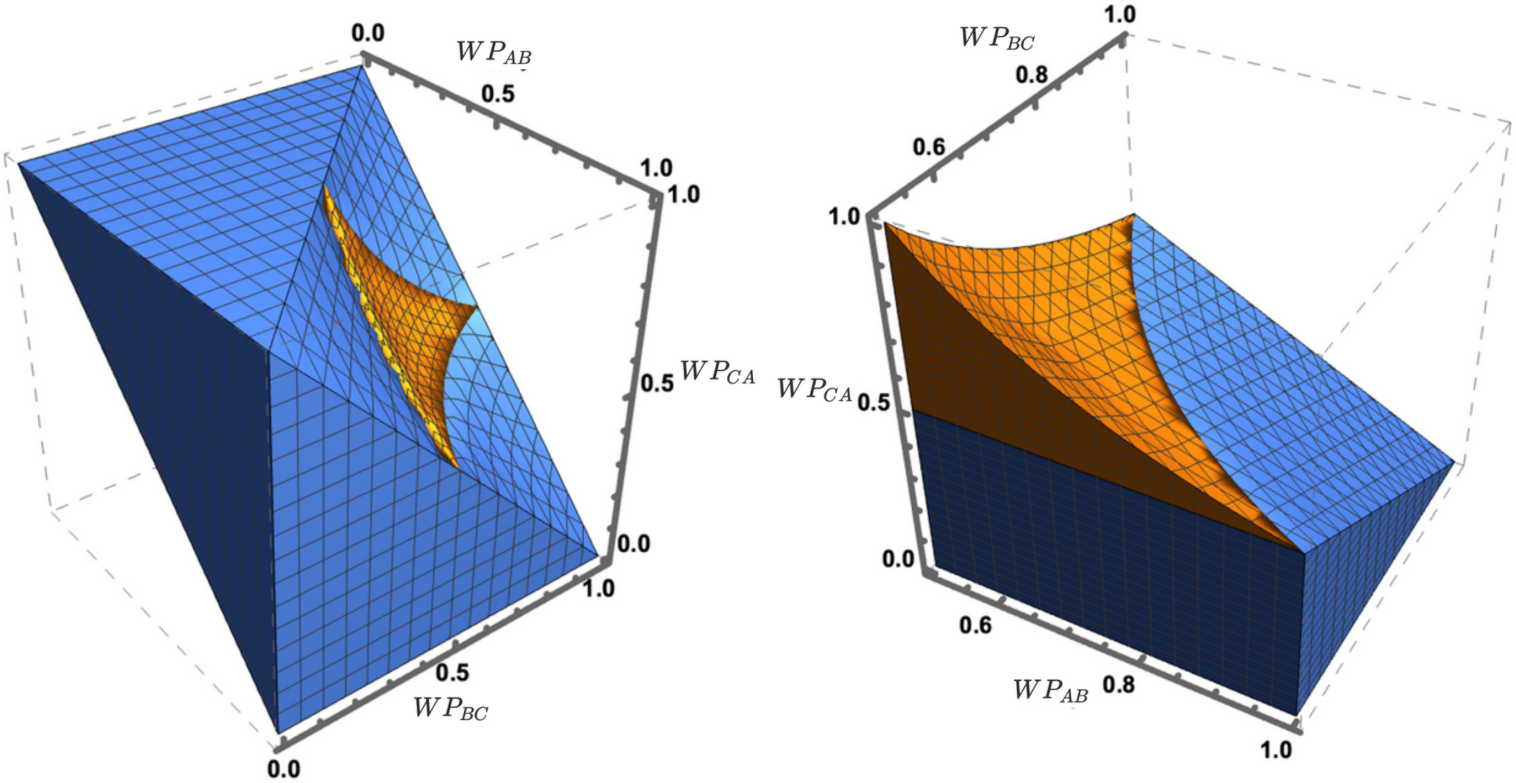
Left: Space $S_{3}$ of all possible triplets of WPs comparing any three independent random variables with blue: Transitive triplets of WPs; orange: Non‐transitive triplets. Right: Space $S_{3}$ restricted to clinically relevant cases when $WP_{\mathrm{AB}}\geq \frac{1}{2}⋂WP_{\mathrm{BC}}\geq \frac{1}{2}$. For example, (A) New Treatment (NT), (B) Standard of Care (SOC), (C) Placebo (P).

**FIGURE A5:**
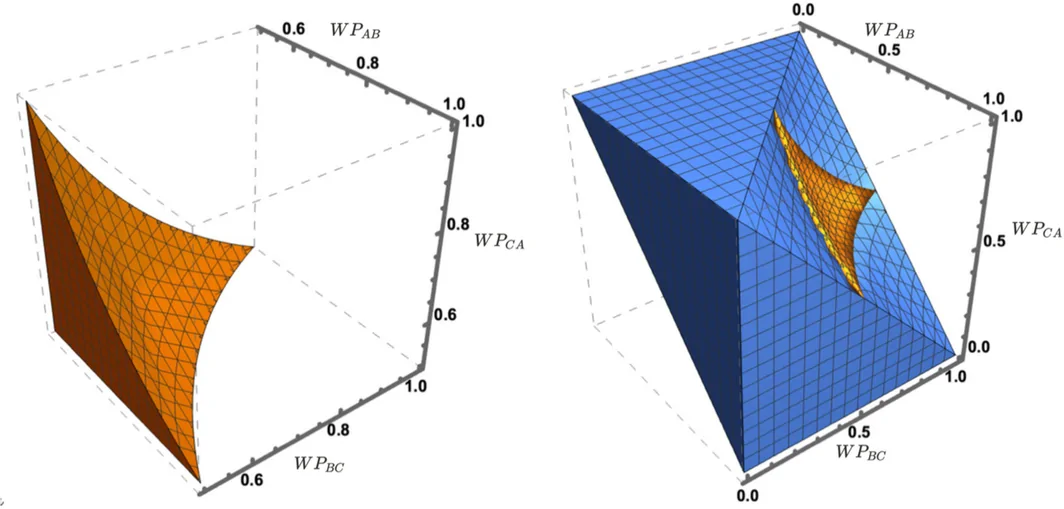
$S_{3}^{o}$ of non‐transitive triplets of WP (left) and space $S_{3}$ (right) of all possible triplets of WP formed by three independent random variables that satisfy Assumptions 1, 2, 3, 4, 1, [Link], 1, 2, 1, [Link], 2, [Link], 3, [Link], 4, [Link], 5, [Link], 6, [Link], [Link], 2, [Link], 8, [Link], 9, [Link], [Link], 11, [Link], 1, 2, 3 Section 2.1.

$S_{3}$ is plotted in Figure 3. Similar plots for the WR are provided in Appendix G: Figure A5, A6. It illustrates that the space of three WPs does not fill out the unit cube. This illustrates why Lemma 7 holds. We will use this in the following case studies.

## 4 Case Studies

The equipoise principle does not allow direct comparison of a novel treatment to placebo when a standard of care treatment is available. We showed that although the WR or WP of novel treatment shows superiority over standard of care, the superiority of novel treatment over placebo cannot be taken for granted. Also, we noted that the space of WPs generated by any three independent random variables does not fill out the unit cube. Therefore, some combinations of WPs are not possible. In general, WPs and WRs are bounded by conditions stated in Lemma 7. In this section, we apply this result to rule out non‐transitivity in the two case studies. In Case Study 1, we also use Statement 2 to confirm that HR, WP, and WR are transitive. Together, these examples illustrate how our framework can be used to establish the superiority of a novel treatment over placebo when a direct comparison is not possible.

### 4.1 Case Study 1. Establishing Transitivity of Comparison of Dabigatran Versus Warfarin Versus Standard of Care

In the 1990s, warfarin was approved as a standard of care for the prevention of ischemic stroke in patients with atrial fibrillation. Boston Area Anticoagulation Trial for Atrial Fibrillation (BAATAF) N=420 was pivotal in establishing the efficacy of **warfarin (w)** over the **standard of care (soc)** in 1990 [48]. In the early 90s, warfarin became the first‐line treatment for patients with atrial fibrillation. In the 2000s, a novel promising class of drugs was discovered called direct oral anticoagulants (DOAC). Treatment **dabigatran (d)** belongs to the DOAC class of drugs, and in 2009 results of the Randomized Evaluation of Long‐Term Anticoagulation Therapy (RE‐LY) trial comparing **dabigatran (d)** with **warfarin (w)** were published. *N* = 18 113 patients with atrial fibrillation (AF) and an elevated risk of stroke were randomized to dose‐adjusted warfarin or dabigatran 110 mg or 150 mg twice daily [50]. Treatment with dabigatran 150 mg compared with warfarin led to the reduction of the rate of the primary endpoint (stroke or systemic embolism) with ${\hat{\mathrm{HR}}}_{d\;\mathrm{vs}.w}$, 95% CI of 0.66,[0.53,0.82]. A direct comparison of **dabigatran (d)** with **soc available in the 1980s (soc)** could not be performed because of the principle of equipoise. In the following, we will see if the results of this paper can be used to rule out non‐transitivity.

First of all, if the PH assumption holds in both trials, then by Statement 2, we can establish transitivity of HR and WR. However, BAATAF trial did not use Cox regression to report efficacy of the primary endpoint; therefore, we cannot assume that PH assumption (Assumption 4, Section 2.1) holds and subsequently cannot use Statement 2 to rule out non‐transitivity. Instead, we could use Lemma 7. First, we must estimate WR values. We reconstructed WR from published Kaplan Meier survival curves [48]; the details of the reconstruction can be found in Appendix K. Based on our estimations, ${\hat{\mathrm{WR}}}_{w\;\mathrm{vs.}\;\mathrm{soc}}=2.51$. In 1998, testing the PH assumption was recommended by the FDA [51]; hence, if we can assume the PH assumption, we can use Table 1 to estimate the WR with ${W\hat{R}}_{d\;\mathrm{vs.}\;w}=1.52$. By Lemma 7 (2) transitivity is guaranteed if $1.52={\hat{\mathrm{WR}}}_{d\;\mathrm{vs.}\;w}\geq \frac{1+{\hat{\mathrm{WR}}}_{w\;\mathrm{vs.}\;\mathrm{soc}}}{{\hat{\mathrm{WR}}}_{w\;\mathrm{vs.}\;\mathrm{soc}}-1}=2.32$. Unfortunately, observed WR is not high enough and does not meet the threshold from Lemma 7, and therefore, we cannot guarantee transitivity. In this situation, we recommend checking whether Lemmas 2, [Link], 3, [Link], 4, [Link], 5, [Link], 6 apply and transitivity may be established based on the distribution functions of times‐to‐events in three arms. Further details can be found in the Appendix: Note A1: Relaxing censoring Assumption for Lemma 2–6. We can also look for subgroups in which we can guarantee transitivity. Meta‐analysis of RCTs of warfarin versus placebo estimated ${\hat{\mathrm{HR}}}_{w\;\mathrm{vs.}\;\mathrm{soc}\;|\;\text{women}}=0.16$ [52]. Assuming the PH assumption, we have ${\hat{\mathrm{WR}}}_{w\;\mathrm{vs.}\;\mathrm{soc}|\;\text{women}}=6.25$. Using the subgroup analysis of the primary endpoint reported in the forest plot of RE‐LY [50], female participants had ${\hat{\mathrm{HR}}}_{d\;\mathrm{vs.}\;w\;|\;\text{women}}=0.56$ with 95% CI of [0.39,0.80]. Assuming the PH assumption, this means ${\hat{\mathrm{WR}}}_{d\;\mathrm{vs.}\;w\;|\;\text{women}}=1.79.$ If we apply Lemma 7 (2) now, we can guarantee transitivity if $1.79={\hat{\mathrm{WR}}}_{d\;\mathrm{vs.}\;w\;|\;\text{women}}\geq \frac{1+{\hat{\mathrm{WR}}}_{w\;\mathrm{vs.}\;\mathrm{soc}}}{{\hat{\mathrm{WR}}}_{w\;\mathrm{vs.}\;\mathrm{soc}}-1}=1.38$. This holds, thus direct comparison of dabigatran with placebo is guaranteed to be transitive among female participants. These results are summarized in Tables 3A and 3B.

**TABLE 3A:** Case Study 1, part A. $\hat{\text{WR}}=2.51$ was observed in the BAATAF trial (warfarin versus soc arm) and $\hat{\text{WR}}=1.52$ in RE‐LY trial (dabigatran versus warfarin) for stroke or systemic embolism outcome. Column “Transitivity threshold” reports lower threshold of 2.32 on WR. This threshold guarantees transitivity of the direct comparison of novel treatment (dabigatran) with placebo. RE‐LY trial does not meet this threshold, therefore based on the strength of the association of warfarin to the placebo and dabigatran with warfarin, non‐transitivity cannot be ruled out.

Warfarin vs. standard of care (BAATAF trial)			
$\hat{\text{WR}}$	L95% CI	U95% CI	Transitivity threshold
2.51	1.17	6.32	2.32

Dabigatran vs. warfarin (RE‐LY trial)			
$\hat{\text{WR}}$	L95% CI	U95% CI	Meets the transitivity threshold?
1.52	1.22	1.89	No

**TABLE 3B:** Case Study 1, part B. $\hat{\text{WR}}=1.79$ observed in the RE‐LY trial (dabigatran versus warfarin) among female participants. Column “Transitivity threshold” reports lower threshold (of 1.38) that guarantees transitivity of the unobserved comparison. According to Lemma 7, if warfarin is superior to placebo in the cohort of women with WR larger than 1.38, this chain of comparisons is transitive and WR comparing dabigatran with placebo is guaranteed to be > 1.0, thereby obviating the need for direct comparison in this subgroup of participants. Note that 95% confidence interval of [1.25, 2.56] for ${\hat{\text{WR}}}_{d\;\mathrm{vs}.\;w\;|\;\text{women}}=1.79$ was obtained from HR reported in the forest plot in [50] using plot digitizer.

Warfarin vs. standard of care among female participants (meta‐analysis)			
$\hat{\text{WR}}$	L95% CI	U95% CI	Transitivity threshold
6.25	2.22	20.0	1.38

Dabigatran vs. warfarin among female participants (RE‐LY trial)			
$\hat{\text{WR}}$	L95% CI	U95% CI	Meets the transitivity threshold?
1.79	1.25	2.56	Yes

In summary, when the PH assumption holds, Statement 2 is stronger than Lemma 7. However, in the absence of the PH assumption, when we aim to prove the transitivity of WP or WR, Lemma 7 remains a viable alternative.

### 4.2 Case Study 2. Establishing the Transitivity of TAVR Versus SAVR Versus Non‐Surgical Treatment

In 2019, the results of the Transcatheter Aortic‐Valve Replacement with a Balloon‐Expandable Valve (TAVR) in Low‐Risk Patients trial were reported. The TAVR arm was compared to the surgical aortic valve replacement (SAVR) arm. The primary endpoint was death, stroke, or rehospitalization at 1 year. Because some of the components of the primary endpoint show signs of nonproportionality of hazards, and the primary endpoint is hierarchical, authors reported ${\hat{\mathrm{WR}}}_{\text{TAVR}\;\mathrm{vs.}\;\text{SAVR}}=1.88$ **with 95% CI of [1.29, 2.76]** at year 1, indicating a significant treatment effect [53]. In another trial published in 2020, SAVR was compared to conservative non‐surgical care (NS) in asymptomatic patients with severe aortic stenosis [54]. The primary endpoint was death during or within 30 days after surgery or death from cardiovascular causes during the entire follow‐up period. Main trial publication reports ${\hat{\mathrm{HR}}}_{\text{SAVR}\;\mathrm{vs.}\;\mathrm{NS}}=0.09$ **with 95% CI of [0.01, 0.67]** indicating very strong treatment effect (median follow‐up was approximately 6 years). Assuming the PH assumption holds in this trial, therefore HR at year 1 of follow‐up is also 0.09 and it is equivalent to ${\hat{\mathrm{WR}}}_{\text{SAVR}\;\mathrm{vs.}\;\mathrm{NS}}=11.11$. Since ${\hat{\mathrm{WR}}}_{\text{TAVR}\;\mathrm{vs.}\;\text{SAVR}}=1.88$, Lemma 7 (2), implies that the threshold of $WR_{\text{threshold}}=3.27$ guarantees transitivity. Since ${\hat{\mathrm{WR}}}_{\text{SAVR}\;\mathrm{vs.}\;\mathrm{NS}}=11.11>3.27$, transitivity is guaranteed. In this case study, we assume that Assumptions 1, 2, 3 (from Section 2.1) hold and that the results of the two trials are transferable to a three‐arm trial with the same or stronger WRs and/or HRs. In this example, there were signs of violation of the PH assumption in the TAVR/SAVR trial. Had the PH assumption been held in both trials, we could have inferred transitivity using Statement 2. The summary of this trial can be found in Table 3C.

**TABLE 3C:** Case Study 2. In the case study the reported WR of TAVR versus SAVR was $\hat{\text{WR}}=1.88$. This led to a lower threshold of 3.27 that guarantees transitivity. The SAVR versus non‐surgical treatment has a $\hat{\text{WR}}=11.11$, which is above the threshold, thus transitivity was guaranteed.

TAVR vs. SAVR			
$\hat{\text{WR}}$	L95% CI	U95% CI	Transitivity threshold
1.88	1.29	2.76	3.27

SAVR vs. non‐surgical treatment			
$\hat{\text{WR}}$	L95% CI	U95% CI	Meets the transitivity threshold?
11.11	1.49	100	Yes

More examples of data analysis of RCTs are presented in Appendix J: Practical Example A1.

### 4.3 Notes About Case Studies

**Note 1**. Lemma 7 does not assume that the principle of randomization is preserved, nor does it assume the similarity of distributions of the demographic's characteristics in the three arms. Lemma 7 makes only one assumption, namely that observations in trial arms are independent. This condition is usually satisfied unless factorial trial design was used. Therefore, if the product of two WPs is above 0.5, then the relationship is transitive in all generality for all stochastic processes that could have generated these WPs. However, in Lemma 7, we are comparing variables $T_{C}$ to $T_{B}$ and $T_{B}$ to $T_{A}$. Whereas in these case studies, we compared $T_{C}$ to $T_{B_{1}}$ and $T_{B_{2}}$ to $T_{A}$ to infer the strength of the direct comparison of $T_{C}$ versus $T_{A},$ where $T_{B_{1}}$ and $T_{B_{2}}$ are two arms in two different trials of the same drug B. Therefore, one additional assumption that we had to make is that ${\mathrm{WR}}_{A\;\mathrm{vs.}\;B_{1}}={\mathrm{WR}}_{A\;\mathrm{vs.}\;B_{2}}$ and ${\mathrm{WR}}_{B_{1}\;\mathrm{vs.}\;C}={\mathrm{WR}}_{B_{2}\;\mathrm{vs.}\;C}$. This is milder than the assumption that two event‐generating processes (in $T_{B_{1}}$ and $T_{B_{2}}$) are identical (IID). Indeed, Lemma 7 does not assume anything about the shape of distribution functions. It relies on the strength of the comparisons (WPs, WRs, or HRs) and Assumptions 1, 2, 3, (Section 2.1).

**FIGURE 4:**
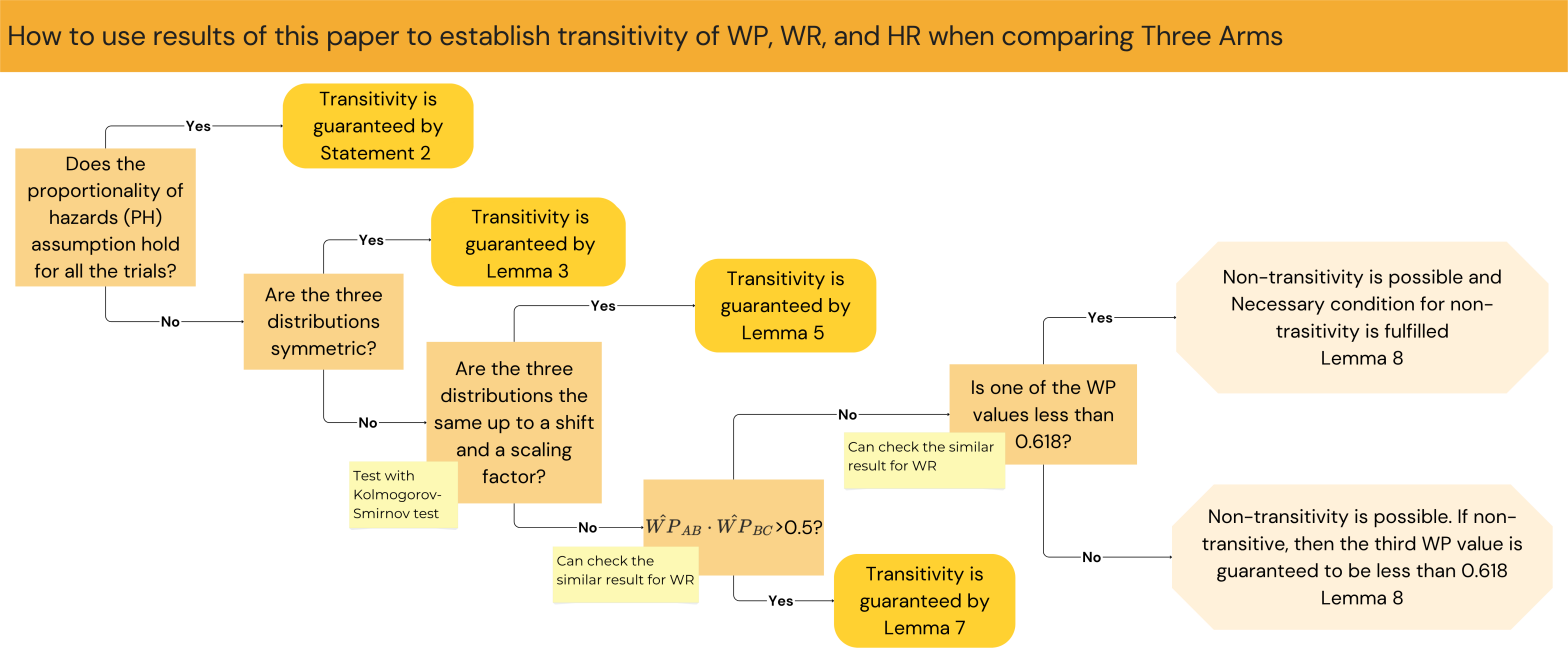
Summary of methods.

**Note 2**. In the case studies and practical guide (Figure 4), we do not observe the true values directly; instead, we rely on their estimates. Provided these estimates are sufficiently accurate, the procedures we present remain valid. We also note that to test the assumptions underlying our lemmas, we can use Appendix: Note A1: Relaxing censoring Assumption for Lemma 2–6 or standard statistical testing methods.

## 5 Discussion and Outlook

### 5.1 Main Results

In this work, we examine the long‐run non‐transitive behavior of the WR, the WP, and the HR. We define non‐transitivity as a property of distribution functions of random variables, a long‐run behavior. The same definition was used by Steinhaus, Trybuła, Savage Jr., Efron, Bogdanov, Komisarski, Gillen, Lumley, Poddiakov, and others [19, 26, 27, 29, 30, 39, 55]. Note that the long‐run non‐transitivity that we study in this paper is different from the lack of transitivity of *realizations* of specific times‐to‐events, studied by several authors [56, 57, 58, 59], including Oakes, who referred to it as “intransitivity.” Here, we investigate how long‐run non‐transitivity can emerge when using WP, WR, and HR to compare time‐to‐event data distributions. Specifically, we show that WR and WP can exhibit paradoxical, non‐transitive loops in pairwise comparisons among three or more independent random variables. As noted by Lumley and Gillen, non‐transitivity leads to at least two problems: (1) identification of the best treatment is not possible, and (2) transitivity cannot be taken for granted [19]. In this work, we answer the question: what are conditions, that guarantee transitivity of WP, WR, and HR when comparing three treatments in randomized controlled trials? In Figure 4, we summarized these conditions. Briefly, non‐transitivity can be ruled out if (a) the PH assumption holds; (b) for certain families of distribution functions (random variables or their monotonic transformations with symmetric pdfs; random variables with the same distributions up to a shift, etc.); (c) if two observed WRs (or WPs) are strong enough. We used these results in Case Studies 1 & 2 to establish the transitivity of three treatments using published clinical trials. The necessary condition for non‐transitivity states that a non‐transitive loop of length n contains at least one WR that is less than or equal to $4\cos^{2}\frac{\pi }{n+2}-1$, and for the WP, this condition is $\mathrm{WP}\leq 1-\frac{1}{4\cos^{2}\frac{\pi }{n+2}}$. When n=3, this boundary for WR is the golden ratio, and for WP it is golden ratio‐1. In the limit, as n, the length of the loop goes to infinity, this boundary is $\frac{3}{4}$ for WP and 3.0 for WR.

### 5.2 What If We Can't Rule Out Non‐Transitivity? Is This a Flaw or a Function of Head‐to‐Head Comparison?

Lumley and Gillen [19] prove that only univariate statistics are transitive under all scenarios, while statistics that are bi‐variate head‐to‐head comparisons can be non‐transitive. Therefore, axiomatically‐transitive statistics include cumulative probability of an event at time T in a specific trial arm, restricted mean survival time [60, 61], means, medians, and other univariate statistics. Bivariate statistics such as WP, WR, WOs, and even log‐rank test [5], can exhibit non‐transitivity. Lumley and Gillen [19, 62] further argue that non‐transitivity in hypothesis testing constitutes a flaw of the employed test statistics, for example, WR. Brunner, Bathke, Konietschke, and others [5, 34, 63, 64] proposed potential solutions to address the non‐transitivity issue in statistical tests like Kruskal‐Wallis and van Elteren, among others. On the other hand, Poddiakov et al. [39, 65] argue that non‐transitivity is a natural phenomenon with practical utility across various fields. In our opinion, when the time to all‐cause death in three randomized arms forms a non‐transitive loop, it signals a potential weakness of the treatment effect, implies ambiguity in trial results, and highlights the absence of a clear superior treatment. This weakness is masked if testing is restricted to purely transitive statistics. We suggest that statistics based on head‐to‐head comparisons may provide a different and complementary evaluation angle, thus warranting their inclusion in analysis.

### 5.3 Implications for Trial Design

Mathematically, the non‐transitivity of WP and WR is largely a property of the shape of the distribution functions of times‐to‐event.

We may ask: what causes such a non‐transitive loop? Explanations include all reasons for violation of the PH assumption, that is, (i) changing dynamics of disease progression due to intervention (e.g., surgery); (ii) late separation of the curves due to delayed treatment effect; (iii) heterogeneity of treatment effect (e.g., treatment is harmful in a subgroup); (iv) treatment switching at progression to a next stage, etc. The first two reasons are informed by the biology of the disease. When (iii) or (iv) are possible, to address (iii), we recommend subgroup analysis as in Case Study 1, or using contemporary approaches to detect subgroups with heterogeneous treatment effect [66]. To address (iv), better monitoring of treatment compliance and inclusion of strategies to mitigate this in the trial protocol.

Summing up these results, fundamental reasons behind non‐transitivity cannot, in general, be resolved with trial design. Instead, we can approach this problem on a case‐by‐case basis and use the results of this manuscript to rule out non‐transitivity for a set of treatments.

### 5.4 Strengths, Limitations, and Future Work

In this paper, we investigate the long‐run non‐transitivity of WR and WP. A major focus of this work is developing practical strategies to identify and rule out non‐transitivity.

In Assumption 2 (Section 2.1), we assumed the absence of censoring. However, this assumption can be relaxed. For simplicity, consider WPs. In this setting, Lemma 7 and the result by Trybuła remain valid as long as a consistent estimator of the WP is available. Uno et al. [67] propose such an estimator, which accounts for censoring through inverse probability of censoring weights. This method yields consistent estimates under independent censoring.

We further assumed that the two random variables take the same values only on a set of measure zero, that the outcomes are simple (i.e., without hierarchical structure), and that the HR is modeled as a function of a single variable—the trial arm. Relaxing these assumptions offers a promising direction for future investigation.

Further work is needed to deepen the understanding of non‐transitivity in medical research. Within RCTs, it is essential to expand the set of conditions that ensure transitivity and explore the effects of censoring, relax the assumption of non‐overlapping values, extend the framework for composite outcomes, and investigate the impact of relaxing independence assumptions. The latter would be especially valuable for extending these results to clinical trials with factorial designs.

To our knowledge, this is the first study that defines conditions that rule out long‐run non‐transitivity of the WP, WR, and HR in RCTs. Our goal is to bring attention to this crucial property and to emphasize that transitivity should not be assumed without verification.

### 5.5 Non‐Transitivity Reports Across Different Fields

Long‐run non‐transitivity has been observed across a range of disciplines, including biology, game theory, theoretical statistics, econometrics, and quantum mechanics [36, 37, 68, 69, 70, 71]. In bacteriology, non‐transitivity is a well‐established phenomenon, playing a key role in maintaining and promoting the diversity of bacteria species [36, 37]. Liao et al. [37] conducted a series of wet‐lab experiments using three different bacterial strains. When the interactions among the strains were transitive, the most toxic strain consistently outcompeted and dominated the other two. However, when the strains formed a non‐transitive loop, the dynamics changed dramatically. In this non‐transitive setup, all three strains were able to coexist. Moreover, depending on their spatial arrangement within the petri dish, the system could exhibit counterintuitive outcomes—such as the survival of the weakest strain or the survival of the strongest strain's adversary. Non‐transitivity is also commonly reported in sports [72], particularly in chess [73, 74]. In econometrics, it is studied in game theory when decisions are made under uncertainty and risk [35]. In quantum mechanics, outcomes of classical measurements are intrinsically probabilistic, and non‐transitivity is also possible, as reported in [68, 75]. Gorbunova and Lebedev [76] study the effect of non‐transitivity in Bayesian queueing models. In medical literature, non‐transitivity is reported in network meta‐analysis and is attributed to heterogeneity of compared populations [77].

## 6 Conclusions

In this paper, we study Efron‐type long‐run non‐transitivity, not to be mixed with intransitivity reported by Oakes and other authors. We demonstrate that the WPs and WRs need not be transitive. Moreover, we derive sufficient conditions ensuring the transitivity of WP, WR, and HR. We also investigate the necessary conditions under which non‐transitivity arises in WP and WR and show that even very high pairwise comparisons may participate in a non‐transitive triplet. We view non‐transitivity as a signal of a weak stochastic relationship between three random variables. Therefore, the possibility of non‐transitivity is not a deficiency of the WP or WR but rather a valuable property and shouldn't be ignored. We argue that we should confront non‐transitivity, and to do so, we provide criteria that rule it out. We note that many statistics, including some that are used to report efficacy in RCTs, are not designed to “detect” non‐transitivity, while metrics that are based on head‐to‐head comparisons (such as WR) can detect non‐transitivity. Therefore, measures of the strength of stochastic relationships may provide complementary information on the efficacy of treatment in clinical trials. We argue that the strength of stochastic relationships should be explicitly assessed and reported to fully understand all aspects of treatment efficacy.

**Nomenclature**
Upper case letters: random variables
Lower case letters: sample realizations of random variables
A,B,C
: three arms in a randomized controlled trial
$T_{A}$
: a random variable representing time‐to‐event in arm A of a randomized controlled trial
$t_{\mathrm{Ai}}$
: time‐to‐event of participant i who was randomized to arm A
Non‐transitivity: long‐run property of random variables compared with head‐to‐head comparisons. This phenomenon was studied in this paper
Intransitivity: property of sample realizations of random variables when it is impossible to determine a winner (see Oakes for details [12, 13]). This phenomenon was not studied in this paper
WP: win proportion (defined in Section 2.2)
WR: win ratio (defined in Section 2.3)
HR: hazard ratio (defined in Section 2.5)
RCT: randomized controlled trial
PH: proportionality of hazards assumption
$S_{n}$
: the space of n win proportions formed by n random variables $T_{1},T_{2},\ldots ,T_{n}:S_{n}=\{{\text{WP}}_{T_{1},T_{2}},{\text{WP}}_{T_{2},T_{3}}\ldots ,{\text{WP}}_{T_{n-1},T_{n}},{\text{WP}}_{T_{n},T_{1}}\}$, where ${\text{WP}}_{T_{i},T_{j}}=P(T_{i}>T_{j}).$
$S_{n}^{o}$
: non‐transitive subspace of $S_{n}$: $S_{n}^{o}=\{{\text{WP}}_{T_{1},T_{2}},{\text{WP}}_{T_{2},T_{3}},\ldots ,{\text{WP}}_{T_{n-1},T_{n}},{\text{WP}}_{T_{n},T_{1}}|{\text{WP}}_{T_{i},T_{j}}>0.5\forall i,j\}$

## Funding

O.V.D. received funding from the NIH NHLBI grant R21HL167173, 5K01HL135342, and Swiss Federal Institute of Technology (ETH, Zurich Switzerland), American Heart Association, 17IGMV33860009, 26SFRNCVHD1592182, 26SFRNPVHD1623048. Anna Győrffy‐Kerekes received PhD funding from the Max Planck ETH Center for Learning Systems (CLS). Ilona Demler received funding from the National Science Foundation, GRFP2023.

## Disclosure

In work unrelated to the current study, O.V.D. is listed as a coinventor on a patent application for a “Method for prediction of future cardiovascular disease risk via analysis of IgG glycome. ” assigned to GENOS d.o.o. and the Brigham and Women's Hospital Inc. Demler received funding from Kowa Research Institute for activities unrelated to current work. All other authors have reported that they have no relationships relevant to the contents of this paper to disclose.

## Conflicts of Interest

The authors declare no conflicts of interest.

## Figures and Tables

**FIGURE A1:**
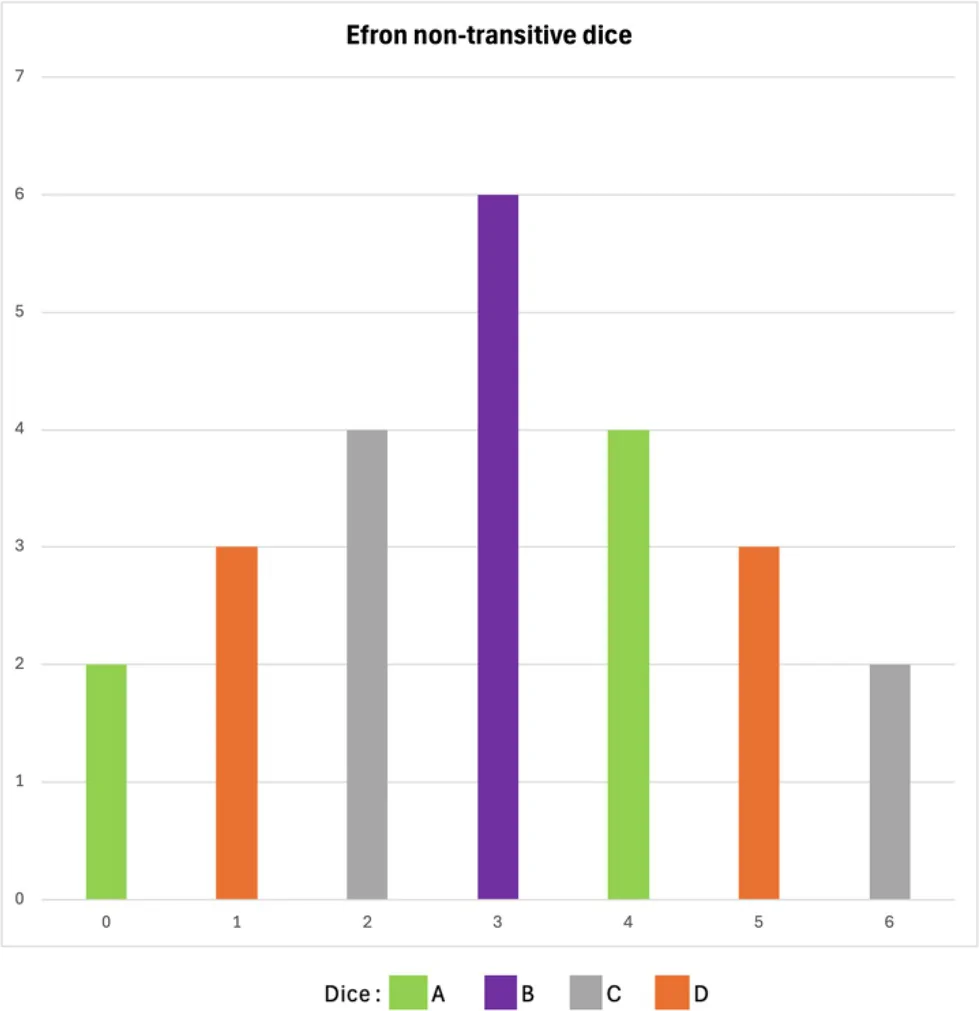
Distribution of scores on Efron non‐transitive dice.

**FIGURE A2:**
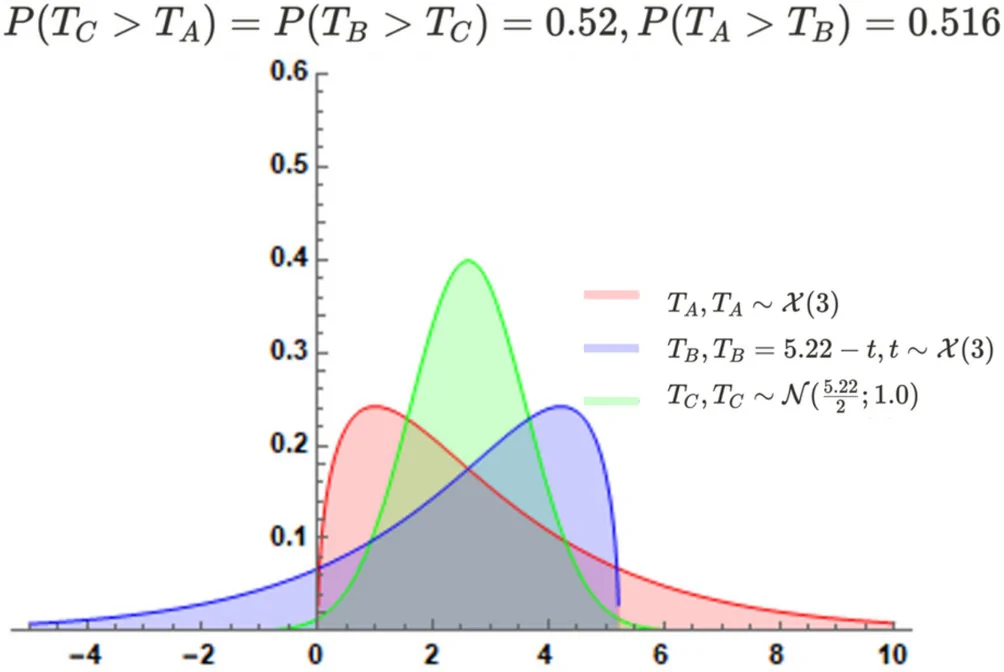
PDFs of three non‐transitive continuous random variables.

**FIGURE A3:**
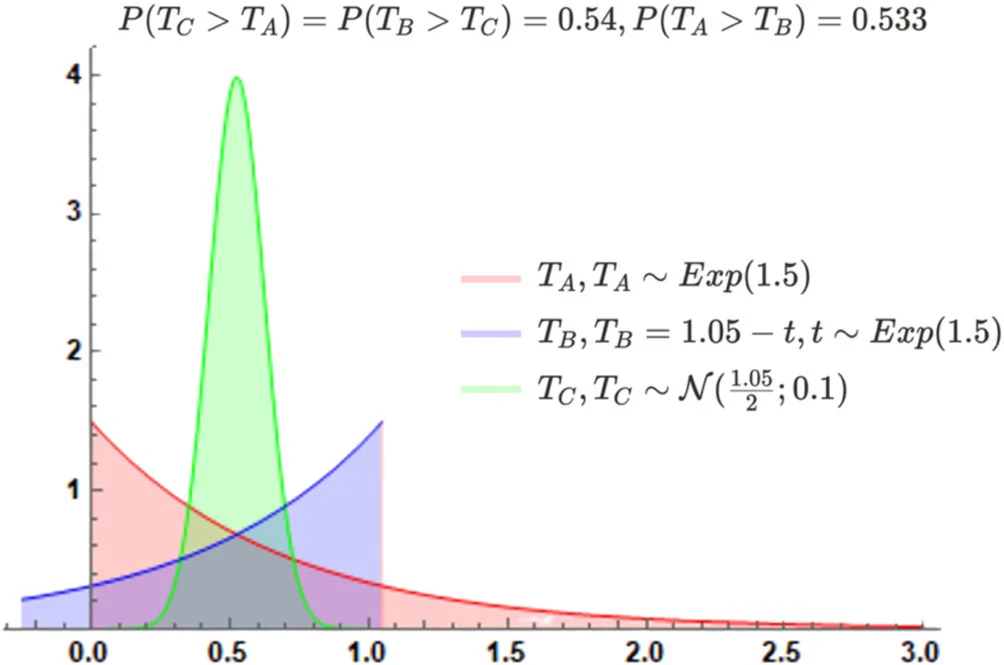
PDFs of three non‐transitive continuous random variables.

**FIGURE A4:**
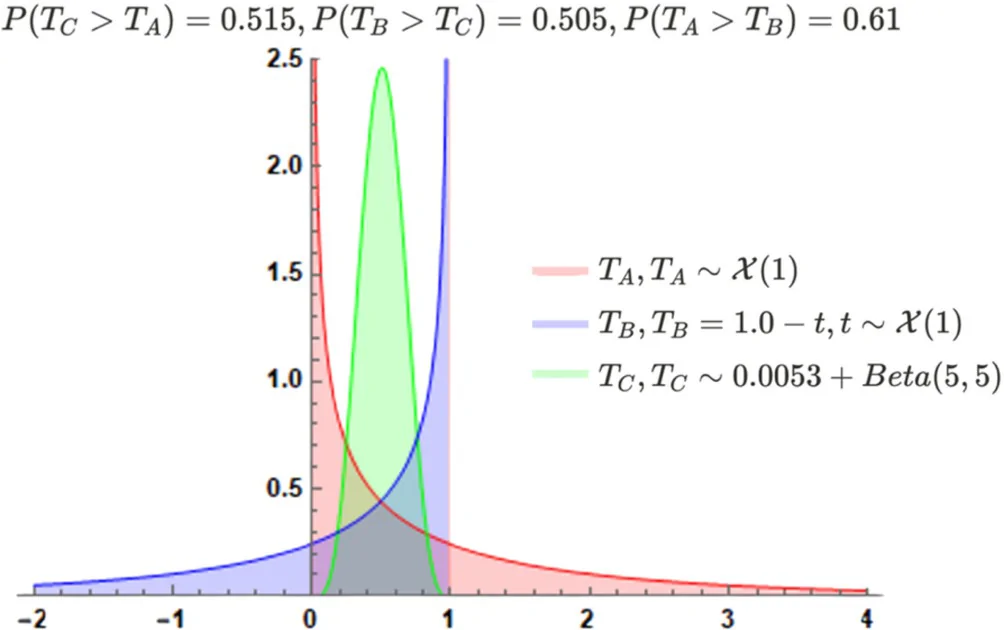
PDFs of three non‐transitive continuous random variables.

**FIGURE A6:**
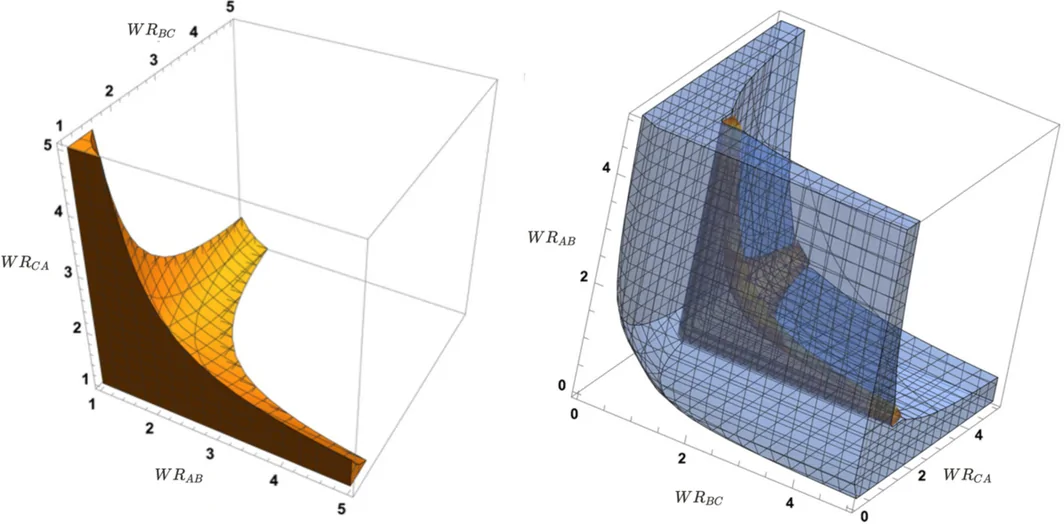
Space of triplets of WRs formed by three independent random variables. *Left:* $S_{3}^{o},$ non‐transitive subspace; right: $S_{3}$. blue: Transitive triplets; yellow: Non‐transitive triplets. Plots are restricted to [1.0; 5.0]^3^ [46] and [0.0; 5.0]^3^ [46].

## Data Availability

The data that support the findings of this study are openly available in Project Data Sphere at https://www.projectdatasphere.org/.

## Appendix A Justification for the Assumptions in Section 2.1

In this paper, we make the following assumptions: Assumption 1
$T_{A},T_{B},T_{C}$ are independent.
Assumption 2 There is no censoring (we assume this for simplicity).
Assumption 3
$P\left(T_{A}=T_{B}\right)=P\left(T_{B}=T_{C}\right)=P\left(T_{C}=T_{A}\right)=0.$

For results involving the hazard ratio, we introduce one additional assumption: Assumption 4 The hazard ratio is constant over time.

**Rationale for these assumptions:** First, Assumption 1 is the independence of trial arms. In randomized clinical trials this is common, and simplifies some of our results. Assumptions 2 and 3 make monotone transformations possible from WP to WR (both for true values and their estimates). Assumption 4 is only assumed for results involving the HR [5, 12, 46]. Theorem 1 in Trybuła, 1961 [27] is important for establishing one of our main results, Lemma 7. Trybuła assumes independence and that any pair of times‐to‐events are distinct if they are observed in two different trial arms. Randomized controlled trials have pre‐specified stopping criteria, after which times‐to‐event are censored (administrative censoring). Therefore, there might be some ties, and we make an assumption of no censoring. We are working on extending the results of this paper to accommodate censoring and the presence of ties. Some initial results of this work are presented in the Appendix: Note A1.

## Appendix B Statement A1: WP, WR, and HR are Strictly Monotonic Transformations of One Another

### Statement A1

Under Assumptions 1, 2, 3 WP, WR, and HR (If Additionally, PH Assumption Holds) Are Strictly Monotonic Transformations of One Another.

WP is used to evaluate efficacy in a randomized controlled trial with two arms. Times‐to‐events in the two arms are denoted as $T_{A}$ and $T_{B}$, and Assumptions 2 and 3 true, so there are no ties. Then, by definition of WP we can write $WP_{\mathrm{AB}}=P\left(T_{A}>T_{B}\right)$, which is estimated with ${\hat{\text{WP}}}_{\mathrm{AB}}=\frac{\sum I\left[t_{\mathrm{Ai}}>t_{\mathrm{Bj}}\right]}{\#\text{comparisons}}=\frac{\sum I\left[t_{\mathrm{Ai}}>t_{\mathrm{Bj}}\right]}{\sum I\left[t_{\mathrm{Ai}}>t_{\mathrm{Bj}}\right]+\sum I\left[t_{\mathrm{Ai}}<t_{\mathrm{Bj}}\right]}.$
Then by definition of WR we can write

$$WR_{\mathrm{AB}}=\frac{P\left(T_{A}>T_{B}\right)}{P\left(T_{A}<T_{B}\right)}=\frac{WP_{\mathrm{AB}}}{1-WP_{\mathrm{AB}}},$$

$${\hat{\text{WR}}}_{\mathrm{AB}}=\frac{\sum I\left[T_{\mathrm{Ai}}>T_{\mathrm{Bj}}\right]}{\sum I\left[T_{\mathrm{Ai}}<T_{\mathrm{Bj}}\right]}=\frac{{\hat{\text{WP}}}_{\mathrm{AB}}}{1-{\hat{\text{WP}}}_{\mathrm{AB}}}.$$

Hence, we have that $WP=\frac{\text{WR}}{1+\text{WR}}$ and $\text{WR}=\frac{\text{WP}}{1-\text{WP}}$ (also true for their estimates). If additionally the assumption of proportionality of hazards holds, then $\mathrm{HR}=\frac{1}{\text{WR}}$. Indeed, this can be shown as follows. (We will only show this for the continuous event time case, where the hazard ratio is utilized). Let us start with two treatments, A and B with hazard rate functions $h_{A}\left(t\right),h_{B}\left(t\right)$ and cumulative hazard functions $H_{A}\left(t\right),H_{B}\left(t\right)$ respectively. If the PH assumption holds, we have the following:

$$h_{A}\left(t\right)=h\left(t\right)\alpha ,H_{A}\left(t\right)=H\left(t\right)\alpha$$

$$h_{B}\left(t\right)=h\left(t\right)\beta ,H_{B}\left(t\right)=H\left(t\right)\beta$$

where h(t) and H(t) are hazard rate and cumulative hazard correspondingly, that is, $\frac{\mathrm{dH}\left(t\right)}{\mathrm{dt}}=h\left(t\right)$. Then the hazard ratio is

$${\mathrm{HR}}_{\mathrm{AB}}=\frac{\beta }{\alpha }.$$

Now we also know, that $h_{A}\left(t\right)=\frac{f_{A}\left(t\right)}{1-F_{A}\left(t\right)}$ and $H_{A}\left(t\right)=-\log\left(1-F_{A}\left(t\right)\right)$, where $f_{A}\left(t\right)$ is the time‐to‐event probability density function and $F_{A}\left(t\right)$ is the cumulative distribution function. Hence, we have

$$f_{A}\left(t\right)=\exp\left(-H_{A}\left(t\right)\right)h_{A}\left(t\right)=\exp\left(-\alpha H\left(t\right)\right)\alpha h\left(t\right)$$

$$F_{A}\left(t\right)=1-\exp\left(-H_{A}\left(t\right)\right)=1-\exp\left(-\alpha H\left(t\right)\right).$$

Similarly, the results also hold for treatment B. Then we can use the integral form of the win proportion to find (if x∼F(·),y∼G(·), then P(x>y) = $E{\left[G\left(x\right)\right]}_{F\left(\cdot \right)}$):

$$\begin{array}{cc} & WP_{\mathrm{AB}}=\int_{0}^{\infty }f_{A}\left(t\right)\left(1-F_{B}\left(t\right)\right)\mathrm{dt}=\int_{0}^{\infty }\exp\left(-\alpha H\left(t\right)\right)\alpha h\left(t\right)\exp\left(-\beta H\left(t\right)\right)\mathrm{dt}\\ & \;=\int_{0}^{\infty }\exp\left(-\left(\alpha +\beta \right)H\left(t\right)\right)\alpha h(t)\mathrm{dt}. \end{array}$$

Let us plug in u=H(t) (t:0→∞,u:0→∞)

$$WP_{\mathrm{AB}}=\int_{0}^{\infty }\exp\left(-\left(\alpha +\beta \right)u\right)\mathrm{\alpha du}={\left[-\frac{\alpha }{\alpha +\beta }\exp\left(-\left(\alpha +\beta \right)u\right)\right]}_{0}^{\infty }=\frac{\alpha }{\alpha +\beta }.$$

Since the hazard ratio is $\frac{\beta }{\alpha }$, we have $HR_{\mathrm{AB}}=\frac{1-WP_{\mathrm{AB}}}{WP_{\mathrm{AB}}}=\frac{1}{WR_{\mathrm{AB}}}$. WR, WP, and HR are monotonic transformations of one another by calculating the derivative of $r\left(x\right)=\frac{x}{1+x}.r^{\prime }\left(x\right)={\left(\frac{x}{1+x}\right)}^{\prime }=\frac{1+x-x}{{\left(1+x\right)}^{2}}=\frac{1}{{\left(1+x\right)}^{2}}>0$ for all real values of x. Thus, we conclude that the WP and WR are monotonic increasing transformations of each other. Under Assumption 4, $\mathrm{HR}=\frac{1}{\mathrm{WR}}$ so, it is a monotonically decreasing function of two other statistics.

## Appendix C Statement A2: Equivalent Non‐Transitive Loops

### Statement A2

*If* $T_{A},\;T_{B},\;T_{C}$ Form a Non‐Transitive Loop Based on One of the Three Measures (WR, WP or HR), Then $T_{A},\;T_{B},\;T_{C}$ Form a Non‐Transitive Loop Based on the Other Two.

If $WP_{\mathrm{AB}}>\frac{1}{2},WP_{\mathrm{BC}}>\frac{1}{2},WP_{\mathrm{CA}}>\frac{1}{2}$, then by Statement A1 this is equivalent to $WR_{\mathrm{AB}}>1,WR_{\mathrm{BC}}>1,WR_{\mathrm{CA}}>1$ and $HR_{\mathrm{AB}}<1,HR_{\mathrm{BC}}<1,HR_{\mathrm{CA}}<1$. Which means $T_{A},\;T_{B},\;T_{C}$ forms a non‐transitive loop for HR and WR as well. Similarly, we could reason the other directions.

## Appendix D Examples of Random Variables That Form Non‐Transitive Loops

### Example A1. Efron Dice: An Example of Non‐Transitive Discrete Random Variables

Example A1 in Figure A1 of four non‐transitive dice was developed by Bradley Efron and popularized by Martin Gardner. Scores 0, 1, 2, 3, 4, 5, 6 are distributed on the sides of four dice A, B, C and D as shown in Figure A1. The score from one roll of a die has a categorical distribution function. It is easy to see that for Efron dice P(A>B)=P(B>C)=P(C>D)=P(D>A)=2/3.

### Example A2

Non‐transitivity of three continuous random variables in Example A2 in Figure A2 can be established by calculating $\mathrm{WP}=P\left(T_{A}>T_{B}\right)=0.516$ $P\left(T_{B}>T_{C}\right)=0.52$ and $P\left(T_{C}>T_{A}\right)=0.52$, using the following formula: If x∼F(·),y∼G(·), then P(x>y) = $E{\left[G\left(x\right)\right]}_{F\left(\cdot \right)}$.

Indeed: $P\left(x>y\right)=\iint_{-\infty y}^{+\infty +\infty }g\left(y\right)f\left(x\right)\text{dxdy}=\int_{-\infty }^{+\infty }g\left(y\right)\int_{y}^{+\infty }f\left(x\right)\text{dxdy}=\int_{-\infty }^{+\infty }\left(1-F\left(y\right)\right)g\left(y\right)\mathrm{dy}=\int_{-\infty }^{+\infty }\left(1-F\left(y\right)\right)\mathrm{dG}\left(y\right)=E{\left[1-F\left(y\right)\right]}_{G\left(\cdot \right)}=\int_{-\infty }^{+\infty }f\left(x\right)\int_{-\infty }^{x}g\left(y\right)\text{dydx}=\int_{-\infty }^{+\infty }G\left(x\right)f\left(x\right)\mathrm{dx}=\int_{-\infty }^{+\infty }G\left(x\right)\mathrm{dF}\left(x\right)=E{\left[G\left(x\right)\right]}_{F\left(\cdot \right)}$.

Examples A3 and A4 can be obtained using the same formula.

### Example A3

Win proportions in Example A3 were evaluated using the relationship P(x>y) = $E{\left[G\left(x\right)\right]}_{F\left(\cdot \right)}$ from Example A1, where x∼F(·),y∼G(·) (Figure A3).

### Example A4

$\mu_{A}=1.0,\mu_{B}=0,\mu_{C}=0.0053+\frac{5}{5+5}=0.5053$.

Win proportions in Example A4 were evaluated using the relationship Pr(x>y)= $E{\left[G\left(x\right)\right]}_{F\left(\cdot \right)}$ from Example A2, where x∼F(·),y∼G(·) (Figure A4).

## Appendix E Sufficient Conditions

### Lemma 1

Three Binary 0‐1 Random Variables Are Always Transitive. 
*Case 1: if ties are split*. We will assume that in the presence of ties, we account for them by splitting ties equally between wins and losses. This updated statistic is known as win odds: Let $P\left(T_{A}=1\right)=p_{A}$ and $P\left(T_{B}=1\right)=p_{B}$, then win odds (WO) is defined as:

$$WO_{\mathrm{AB}}=\frac{P\left(T_{A}>T_{B}\right)+0.5P\left(T_{A}=T_{B}\right)}{P\left(T_{A}<T_{B}\right)+0.5P\left(T_{A}=T_{B}\right)}=\frac{P\left(T_{A}>T_{B}\right)+0.5P\left(T_{A}=T_{B}\right)}{1-P\left(T_{A}>T_{B}\right)-0.5P\left(T_{A}=T_{B}\right)}.$$

WO is a monotonic function of $P\left(T_{A}>T_{B}\right)+0.5P\left(T_{A}=T_{B}\right)$, which is an extension of WP by accommodating ties. We can call it win proportion with ties (WPT). If WPT cannot form non‐transitive loops when comparing three binary variables $T_{A},T_{B}$ and $T_{C}$, then, since WO is a monotonic function of WPT, we will show that the WO also cannot form non‐transitive loops.

$${\mathrm{WPT}}_{\mathrm{AB}}=P\left(T_{A}>T_{B}\right)+0.5P\left(T_{A}=T_{B}\right)=P\left(1,0\right)+0.5\left(P\left(0,0\right)+P\left(1,1\right)\right)=$$

$$=p_{A}\left(1-p_{B}\right)+0.5\left(p_{A}p_{B}+\left(1-p_{A}\right)\left(1-p_{B}\right)\right)=\frac{1+p_{A}-p_{B}}{2}.$$

Therefore ${\mathrm{WPT}}_{\mathrm{AB}}>0.5$ iff $p_{A}>p_{B}$. So non‐transitive loop is not possible.
*Case 2: if ties are ignored*. Let us start with WP:

$${\text{WP}}_{\mathrm{AB}}=P\left(T_{A}>T_{B}\right)=P\left(1,0\right)=p_{A}\left(1-p_{B}\right)>0.5\;\text{or}\;p_{A}>\frac{1}{2\left(1-p_{B}\right)}.$$

Since $p_{A}\leq 1$, $p_{A}\left(1-p_{B}\right)>0.5$ is only possible if $p_{B}<0.5$. Similarly, we also need $p_{A}>0.5$. This means it's only possible if $p_{A}>p_{B}$. Hence a non‐transitive loop is not possible. Now for WR:

$${\text{WR}}_{\mathrm{AB}}=\frac{P\left(T_{A}>T_{B}\right)}{P\left(T_{A}<T_{B}\right)}=\frac{p_{A}\left(1-p_{B}\right)}{\left(1-p_{A}\right)p_{B}}>1\;\text{or}\;p_{A}>p_{B}.$$

Hence a non‐transitive loop is not possible.

### Lemma 2

Three Normally Distributed Random Variables Are Always Transitive. We will show that if $T_{A}∼N(\mu_{A},\;\sigma ),\;T_{B}∼N(\mu_{B},\;\tau )$, then $P(T_{A}>T_{B})\geq 0.5$ iff $\mu_{A}>\mu_{B}$. Indeed, $P(T_{A}>T_{B})=P(T_{A}-T_{B}>0),T_{A}-T_{B}∼N(\mu_{A}-\mu_{B},\sqrt{\sigma^{2}+\tau^{2}})$, so $P\left(T_{A}-T_{B}>0\right)=1-\Phi (\frac{\left(\mu_{A}-\mu_{B}\right)}{\;\sqrt{\left(\sigma^{2}+\tau^{2}\right)}})$. Therefore $P(T_{A}>T_{B})>\frac{1}{2}$ iff $\mu_{A}>\mu_{B}$. Because the comparison of means is always transitive, we conclude that three Normally distributed random variables are always transitive.

### Lemma 3

Three Log‐Normally Distributed Random Variables Are Always Transitive. 
$P\left(T_{A}>T_{B}\right)$ is invariant to monotonic transformation. Therefore $P\left(T_{A}>T_{B}\right)=$ $P\left(e^{T_{A}}>e^{T_{B}}\right)$ and we can use Lemma 2 to infer transitivity.

### Lemma 4

Three Continuous Random Variables With Symmetric Pdfs Are Always Transitive. In other words, $P\left(T_{A}-T_{B}>0\right)>\frac{1}{2}$ if and only if $M_{A}>M_{B}$, where $M_{A},M_{B}$ are points of symmetry of independent random variables $T_{A}$ and $T_{B}$ and $T_{A},T_{B}\in \mathbb{R}$. First, let us prove that if $P\left(T_{A}-T_{B}>0\right)>\frac{1}{2}⟹$ $M_{A}>M_{B}.$ We note that random variables $T_{A}$ and $2M_{A}-T_{A}$ follow the same distribution. Similarly, random variables $T_{B}$ and $2M_{B}-T_{B}$ follow the same distribution. Then we can write:

$$\frac{1}{2}<P\left(T_{A}-T_{B}>0\right)=P\left(T_{A}>T_{B}\right)=P\left(2M_{A}-T_{A}>2M_{B}-T_{B}\right)$$

$$\begin{array}{cc} & =P\left(T_{B}-T_{A}>2\left(M_{B}-M_{A}\right)\right)=P\left(T_{A}-T_{B}<2\left(M_{A}-M_{B}\right)\right)\\ & \;=1-P\left(T_{A}-T_{B}<2\left(M_{A}-M_{B}\right)\right). \end{array}$$

In other words, $1-P\left(T_{A}-T_{B}<2\left(M_{A}-M_{B}\right)\right)>\frac{1}{2}$ or equivalently, $P\left(T_{A}-T_{B}<2\left(M_{A}-M_{B}\right)\right)<\frac{1}{2}$. The two inequalities $P\left(T_{A}-T_{B}>0\right)>\frac{1}{2}$ and $P\left(T_{A}-T_{B}<2\left(M_{A}-M_{B}\right)\right)<\frac{1}{2}$, imply that $2\left(M_{A}-M_{B}\right)>0$. Next, we prove that $M_{A}>M_{B}⟹P\left(T_{A}-T_{B}>0\right)>\frac{1}{2}$. We will do this by contradiction. Define $X=P\left(T_{A}-T_{B}>0\right)$. Then $1-X=1-P\left(T_{A}-T_{B}>0\right).$ As before, we have that

$$1-P\left(T_{A}-T_{B}>0\right)=1-P\left(2M_{A}-T_{A}>2M_{B}-T_{B}\right)$$

$$=1-P\left(T_{B}-T_{A}>2\left(M_{B}-M_{A}\right)\right)=P\left(T_{A}-T_{B}<2\left(M_{A}-M_{B}\right)\right).$$

Suppose that $X<\frac{1}{2}$. Since cumulative probability is defined as the integral of a non‐negative function, and $M_{A}>M_{B}⟹2\left(M_{A}-M_{B}\right)>0$, we have that

$$P\left(T_{A}-T_{B}<2\left(M_{A}-M_{B}\right)\right)<P\left(T_{A}-T_{B}>0\right).$$

This gives us that:

$$\frac{1}{2}\leq 1-X=P\left(T_{A}-T_{B}<2\left(M_{A}-M_{B}\right)\right)<P\left(T_{A}-T_{B}>0\right)=X<\frac{1}{2}$$

$\frac{1}{2}\leq 1-X<X<\frac{1}{2}$, which is a contradiction.

When we completed this manuscript, we became aware of Lebedev [18] proof of this lemma.

Normal, Uniform [−a;a],a>0; Student's t, Logistic and Cauchy distributions have symmetric probability density functions and are therefore always transitive. However, they can still form non‐transitive loops, but only with random variables with asymmetric pdfs such as Chi‐squared, exponential beta.

### Lemma 7

We state this lemma in two forms. For any three random variables $T_{A},T_{B}$ and $T_{C}$ such that Assumptions (1, 2, 3) (Section 2.1) are satisfied,

(1) if ${\mathrm{WP}}_{\mathrm{AB}}\geq \frac{1}{2{\mathrm{WP}}_{\mathrm{BC}}}$, then $T_{A},T_{B}$ and $T_{C}$ are transitive, that is ${\mathrm{WP}}_{\mathrm{AC}}\geq \frac{1}{2}$.

(2) if ${\mathrm{WR}}_{\mathrm{BC}}\geq \frac{1+{\mathrm{WR}}_{\mathrm{AB}}}{{\mathrm{WR}}_{\mathrm{AB}}-1},$ then $T_{A},T_{B}$ and $T_{C}$ are transitive, that is ${\mathrm{WR}}_{\mathrm{AC}}\geq 1$. The Corollary 1.4 of Theorem 1 in Trybuła 1961 [27] states, that for any triplet of independent random variables $T_{A},T_{B}$ and $T_{C}$, ${\text{WP}}_{\mathrm{AB}}\cdot {\text{WP}}_{\mathrm{BC}}\cdot {\text{WP}}_{\mathrm{CA}}\leq \frac{1}{4}$ always holds. Suppose ${\text{WP}}_{\mathrm{AB}}\cdot {\text{WP}}_{\mathrm{BC}}\geq \frac{1}{2}$, then $\frac{1}{2}{\text{WP}}_{\mathrm{CA}}\leq {\text{WP}}_{\mathrm{AB}}\cdot {\text{WP}}_{\mathrm{BC}}\cdot {\text{WP}}_{\mathrm{CA}}\leq \frac{1}{4}$ and therefore ${\text{WP}}_{\mathrm{CA}}\leq \frac{1}{2}$, so $WP_{\mathrm{AC}}\geq \frac{1}{2}$. If we plug in $\mathrm{WP}=\frac{\mathrm{WR}}{1+\mathrm{WR}}$, we obtain (2).

In the corollary to Theorem 1 (equation 12), Trybuła, 1965 [28] proved that for any number of independent random variables $T_{A_{1}},\ldots ,T_{A_{n}}$, the inequality ${\text{WP}}_{A_{1}A_{2}}\cdot {\text{WP}}_{A_{2}A_{3}}\cdot \ldots \cdot {\text{WP}}_{A_{n}A_{1}}\leq \frac{1}{4}$ always holds and therefore Lemma 7 can be extended to longer loops. Furthermore, $\frac{1}{4}$ is a sharp upper bound in the sense that there are sets of independent random variables for which ${\text{WP}}_{A_{1}A_{2}}\cdot {\text{WP}}_{A_{2}A_{3}}\cdot \ldots \cdot {\text{WP}}_{A_{n}A_{1}}=\frac{1}{4}$. Thus, for three random variables, this implies that if ${\text{WP}}_{\mathrm{AB}}\cdot {\text{WP}}_{\mathrm{BC}}>\frac{1}{2}$ then ${\text{WP}}_{\mathrm{CA}}<\frac{1}{2}$ for the inequality to hold.

### Statement 2

If Additionally, Assumption 4 (Section 2.1) Holds for All Comparisons, Then HRs Are Transitive. Let us start with three treatments, treatment A, B, C with hazard functions $h_{A\left(t\right)},h_{B\left(t\right)},h_{C\left(t\right)}$ respectively. If the PH (Proportionality of Hazards) assumption holds, we have the following:

$$h_{A}\left(t\right)=h\left(t\right)\alpha$$

$$h_{B}\left(t\right)=h\left(t\right)\beta$$

$$h_{C}\left(t\right)=h\left(t\right)\gamma ,$$

where h(t) is the common time dependent component. Then the hazard ratios are.

$${\text{HR}}_{\mathrm{AB}}=\frac{\beta }{\alpha };{\text{HR}}_{\mathrm{BC}}=\frac{\gamma }{\beta };{\text{HR}}_{\mathrm{AC}}=\frac{\gamma }{\alpha }.$$

If $\frac{\beta }{\alpha }<1$ and $\frac{\gamma }{\beta }<1$, then $\frac{\gamma }{\alpha }=\frac{\gamma }{\beta }\cdot \frac{\beta }{\alpha }<1\cdot 1=1$. Hence, we have transitivity.

## Appendix F Necessary Conditions

### Lemma 8

Suppose three random variables $T_{A},T_{B}$ and $T_{C}$ satisfy assumptions (1, 2, 3), Section 2.1. If they form a non‐transitive loop, then the following hold:

*(1) At least one of their WPs is less than or equal to* $\frac{\sqrt{5}-1}{2}\approx 0.618$, that is, $\underset{{S\in S}_{n}^{o}}{\sup}\;\min_{\text{WP}\in S}\text{WP}=\frac{\sqrt{5}-1}{2}$.

*(2) At least one of their WRs is less than or equal to* $\frac{\sqrt{5}-1}{3-\sqrt{5}}\approx 1.618$. Bogdanov‐Komisarski result states that $\underset{S\in S_{n}^{o}}{\sup}\;\min_{\text{WP}\in S}\text{WP}=1-\frac{1}{4\cos^{2}\frac{\pi }{n+2}}$ where $S_{n}^{o}$ is the space of sets of non‐transitive WPs of n independent random variables [29, 30]. For n=3, $\underset{S\in S_{3}^{o}}{\sup}\;\min_{\text{WP}\in S}\text{WP}=1-\frac{1}{4\cos^{2}\frac{\pi }{5}}=\frac{\sqrt{5}-1}{2}$. The continuous case follows from Lemma 2 in Trybuła, 1961 [27] which states that the WP of continuous random variables can be approximated with the WP of discrete random variable as closely as possible. (2) follows by using monotonic transformations: WR=WP/(1−WP).

### Lemma 10

$\underset{S\in S_{n}^{o}}{\sup}\;\max_{\text{WP}\in S}\text{WP}=1$. It is enough to show that the following triplet of WPs (0.5,1.0,0.5) is in $S_{3}$. Which can be checked by using characterization of $S_{3}$ from Tybula, 1961 Theorem 1 [27] provided also in Appendix: Trybuła Theorem 1. Indeed, since 0.5+1.0>1.0, we need α(0.5,1.0)≥0.5≥0, where α(·,·) is defined in Appendix: Trybuła Theorem 1. α(0.5,1.0)=0.5, hence, we conclude that $\underset{S\in S_{3}}{\sup}\;\max_{\mathrm{WP}\in S}\text{WP}=1$.

## Appendix G Characterization of $S_{3}$ and $S_{3}^{o}$

In the next lemmas, we show alternative characterizations of $S_{3}$ and $S_{3}^{o}.$

### Lemma 11

$S_{3}$, Space of triplets of WPs formed by any three independent random variables under assumptions (1, 2, 3), Section 2.1, can be described as

$$S_{3}=({⋃}_{A,B,Ccycl}\{WP:WP_{AB}\cdot WP_{BC}<1‐WP_{CA}\})$$

$$⋂\left(\underset{A,B,C\;\text{cycl}}{⋃}\left\{\text{WP}:\left(1-WP_{\mathrm{AB}}\right)\cdot \left(1-WP_{\mathrm{BC}}\right)<WP_{\mathrm{CA}}\right\}\right),$$

$$\;S_{3}^{o}=S_{3}⋂\left\{\text{WP}:{\text{WP}}_{\mathrm{AB}}\geq \frac{1}{2},{\text{WP}}_{\mathrm{BC}}\geq \frac{1}{2},{\text{WP}}_{\mathrm{CA}}\geq \frac{1}{2}\right\},$$

where “A,B,Ccycl” means we permute them cyclically (A,B,C; B,C,A; C,A,B). For any three independent random variables that satisfy Assumptions (1, 2, 3, 4, 1, [Link], 1, 2, 1, [Link], 2, [Link], 3, [Link], 4, [Link], 5, [Link], 6, [Link], [Link], 2, [Link], 8, [Link], 9, [Link], [Link], 11, [Link], 1, 2, 3), Section 2.1, let us denote their WPs as $WP_{\mathrm{AB}}=a,WP_{\mathrm{BC}},=b,WP_{\mathrm{CA}}=c$, $S_{3}=\left\{a,b,c\right|\text{Assumptions}\;\left(1\right)-\left(3\right),\text{section}\;2.1\;\text{hold}\}$. To complete our proof, we need three results. The first is Trybuła, 1961 Theorem 1. We state it explicitly later in Appendix: Trybuła Theorem 1. However, the characterization that follows from this theorem is the following:

$$\begin{array}{cc} & S_{3}=\left\{WP:\left(1-\alpha \left(1-WP_{\mathrm{AB}},1-WP_{\mathrm{BC}}\right)\right)\leq WP_{\mathrm{CA}}\leq \alpha \left(WP_{\mathrm{AB}},WP_{\mathrm{BC}}\right)\right\}\\ & \;=\left\{a,b,c|\left(1-\alpha \left(1-a,1-b\right)\right)\leq c\leq \alpha \left(a,b\right)\right\},\;\left(T1\right). \end{array}$$

where

$$\forall x,y\in \left[0,1\right]\;\alpha \left(x,y\right)=\left\{\begin{array}{ll} \max\left(\frac{1-x}{y},\frac{1-y}{x},1-\mathrm{xy}\right) & \text{for}\;x+y>1\\ 1 & \text{for}\;x+y\leq 1 \end{array}\right..$$

To conclude, we also need the following inequality lemmas:
**Inequality Lemma 11.1**
If a,b,c∈[0,1]

(A1)
c≤α(a,b)

is equivalent to: at least one of the following three inequalities is true

(A2)
ac≤1−b

(A3)
ab≤1−c

(A4)
bc≤1−a

**Inequality Lemma 11.2**
If a,b,c∈[0,1]

1−α(1−a,1−b)≤c

is equivalent to: at least one of the following three inequalities is true

(1−a)(1−c)≤b

(1−b)(1−c)≤a

(1−a)(1−b)≤c.

The proof of Lemmas 11.1 and 11.2 can be found below (Appendix: Inequality Lemma 11.1 and Inequality Lemma 11.2). Putting together characterization T1 and Inequality Lemma 11.1 and Inequality Lemma 11.2 gives us the characterization of $S_{3}$ in Lemma 11. Finally, to get the characterization of $S_{3}^{o}$ from Lemma 11 we just need to use its definition. This concludes our proof of Lemma 11. Now we prove Inequality Lemma 11.1 and Inequality Lemma 11.2.

### Inequality Lemma 11.1

If a,b,c∈[0,1]

(A1)
c≤α(a,b)

is equivalent to: at least one of the following three inequalities is true

(A2)
ac≤1−b

(A3)
ab≤1−c

(A4)
bc≤1−a

*Case 1:* a+b≤1. Then c<1 is always true, so we just need to show that at least one of the (A2), (A3), (A4) inequalities also always holds. If a=0 or b=0, then Equation (A2) or (A4) is true. So we can assume a,b≠0. Let us rephrase the three inequalities

$$\begin{array}{cc} \mathrm{ac} & \leq 1-b\Leftrightarrow c\leq \frac{1-b}{a}\\ \mathrm{ab} & \leq 1-c\Leftrightarrow c\leq 1-\text{ab}\\ \text{bc} & \leq 1-a\Leftrightarrow c\leq \frac{1-a}{b}. \end{array}$$

Then $\frac{1-b}{a}\geq 1\Leftrightarrow 1\geq a+b$, which is true. Thus Equation (A2) always holds.
*Case 2:* a+b>1. Then we have a,b>0. Hence the rephrasing from Case 1 also hold. Hence, we have $c\leq \alpha \left(a,b\right)\Leftrightarrow c\leq \max\left(\frac{1-b}{a},\frac{1-a}{b},1-\mathrm{ab}\right)$ which is equivalent to that at least one of the three rephrased equations is true. Thus we can conclude Inequality Lemma 11.1.

### Inequality Lemma 11.2

If a,b,c∈[0,1]

1−α(1−a,1−b)≤c

is equivalent to: at least one of the following three inequalities is true

(1−a)(1−c)≤b

(1−b)(1−c)≤a

(1−a)(1−b)≤c

The proof follows from plugging in $\tilde{a}=1-a,\tilde{b}=1-b,\tilde{c}=1-c$ into Inequality Lemma 11.1.

## Appendix H Original Results From Trybuła, 1961 [27]

Theorem 1, Trybuła, 1961 plays an important role in the results of this paper. Indeed, both case studies rely on Lemma 7 which follows from Corollary 1.4, Trybuła, 1961. Corollary 1.4 in turn follows from Theorem 1 of the same paper. We restate Theorem 1 below as originally published in Trybuła, 1961 [27].

### Trybuła Theorem 1

*If* $T_{A}$ *,* $T_{B}$ *,* $T_{C}$ Are Independent Random Variables and $P\left(T_{A}=T_{B}\right)=P\left(T_{B}=T_{C}\right)=P\left(T_{C}=T_{A}\right)=0,$

Then we have

1−α(1−ϵ,1−η)≤ζ≤α(ϵ,η),

where $\epsilon =P\left(T_{A}>T_{B}\right),\eta =P\left(T_{B}>T_{C}\right),\zeta =P\left(T_{C}>T_{A}\right)$ and

$$\alpha \left(x,y\right)=\left\{\begin{array}{ll} \max\left(\frac{1-x}{y},\frac{1-y}{x},1-\mathrm{xy}\right) & \text{if}\;x+y>1\\ 1 & \text{if}\;x+y\leq 1 \end{array}\right..$$

In Trybuła, 1961 [27].

In Theorem 1 Trybuła fully defines the space of win proportions for any three random variables that satisfy Assumptions (1, 2, 3) (Section 2.1). This space is plotted in Figure 3. Note that Theorem 1 uses minimal set of assumptions and is true for any distribution function as long as Assumptions (1, 2, 3) (Section 2.1) are satisfied. Note that even under such minimal assumptions, the space of win proportions does not fill the unit cube. Therefore, there are combinations of win proportions that are algebraically impossible. In Lemma 7 we use Corollary 1.4, Trybuła, 1961 to establish transitivity of WP and WR. In case studies 1 and 2 we illustrate how Lemma 7 can be used to rule out non‐transitivity for the third comparison using results of randomized controlled trials.

Corollary 1.4, Trybuła, 1961 is important for Lemma 7 and the case studies. Trybuła states that proof is “analogous to the proof of Corollary 1.3.” For the sake of completeness, we restate Corollary 1.4 below as originally published in Trybuła, 1961 and present its full proof.

### Corollary 1.4

(Trybuła 1961 [27]): Let $T_{A},T_{B}$ and $T_{C}$ Be Independent Random Variables Such That

$$P\left(T_{A}=T_{B}\right)=P\left(T_{B}=T_{C}\right)=P\left(T_{C}=T_{A}\right)=0.$$

Then we have

$$P\left(T_{A}>T_{B}\right)P\left(T_{B}>T_{C}\right)P\left(T_{C}>T_{A}\right)\leq \frac{1}{4}.$$

Let us start with the notation of Trybuła Theorem 1 (above in Appendix: Trybuła Theorem 1), namely:

$$P\left(T_{A}>T_{B}\right)=\epsilon ,P\left(T_{B}>T_{C}\right)=\eta ,P\left(T_{C}>T_{A}\right)=\zeta .$$

Then by Trybuła Theorem 1 (above), we have:
1−α(1−ϵ,1−η)≤ζ≤α(ϵ,η), where

$$\alpha \left(\eta ,\epsilon \right)=\left\{\begin{array}{ll} \begin{array}{l} \max\left(\frac{1-\eta }{\epsilon },\frac{1-\epsilon }{\eta },1-\eta \epsilon \right)\\ 1 \end{array} & \begin{array}{l} \text{if}\;\epsilon +\eta >1\\ \text{if}\;\epsilon +\eta \leq 1 \end{array} \end{array}\right..$$

Hence, we obtain the following:

ϵηζ≤ϵηα(ϵ,η),(Ineq.A)

*Case 1:* ϵ+η≤1: By the inequality between the arithmetic and geometric mean and that 0≤ζ≤1, we have:

$$\sqrt{\eta \epsilon }\leq \frac{\eta +\epsilon }{2}\leq \frac{1}{2}⟹\eta \epsilon \leq \frac{1}{4}⟹\zeta \eta \epsilon \leq \frac{1}{4}.$$

*Case 2:* ϵ+η>1: By the inequality between the arithmetic and geometric mean and that 0≤ϵ,η≤1 we obtain the following inequalities:

$$\sqrt{\left(1-\eta \right)\eta }\leq \frac{1}{2}\Rightarrow \left(1-\eta \right)\eta \leq \frac{1}{4}\Rightarrow \frac{1-\eta }{\epsilon }\eta \epsilon \leq \frac{1}{4},$$

$$\sqrt{\left(1-\epsilon \right)\epsilon }\leq \frac{1}{2}\Rightarrow \left(1-\epsilon \right)\epsilon \leq \frac{1}{4}\Rightarrow \frac{1-\epsilon }{\eta }\epsilon \eta \leq \frac{1}{4},$$

$$\sqrt{\left(1-\eta \epsilon \right)\eta \epsilon }\leq \frac{1}{2}\Rightarrow \left(1-\eta \epsilon \right)\eta \epsilon \leq \frac{1}{4}.$$

Finally, by these equations and (Ineq. A) we have:

$$\epsilon \eta \zeta \leq \epsilon \eta \alpha \left(\epsilon ,\eta \right)=\max\left(\frac{1-\eta }{\epsilon }\eta \epsilon ,\frac{1-\epsilon }{\eta }\epsilon \eta ,\left(1-\eta \epsilon \right)\eta \epsilon \right)\leq \frac{1}{4}$$

and this completes the proof.

### Assumptions of Trybuła Theorem 1

1. $T_{A},T_{B},T_{C}$ are independent random variables.
2. $P\left(T_{A}=T_{B}\right)=P\left(T_{B}=T_{C}\right)=P\left(T_{C}=T_{A}\right)=0$

Assumption 1 (Section 2.1) from the original paper ensures that the first Trybuła assumption holds [27] and Assumption 3 (Section 2.1) from the original paper ensures that the second Trybuła assumption holds. Because we translate Trybuła results to times‐to‐event, we had to add one more assumption of no censoring. How to accommodate censoring is the topic of our current research, but in Note A1 below in Appendix: Note A1: Relaxing Censoring Assumption for Lemma 2–6 we discuss how to extend Lemmas 1–6 of this paper to censored data.

## Appendix I Note A1: Relaxing Censoring Assumption for Lemma 2–6

To apply Lemma 2–6, it is necessary to assess the distributions involved. Normality in censored data can be tested using the method described in [78], which also provides a means for testing lognormality. Symmetry under censoring can be evaluated following the approach outlined in Bacci et al. [79]. Finally, to determine whether two censored datasets arise from the same distribution up to a shift and scale, we can proceed by first applying the Kaplan–Meier estimator to account for censoring [80], then aligning the datasets through appropriate scale and shift adjustments, and finally conducting a Kolmogorov–Smirnov test [81] to assess goodness of fit.

## Appendix J Practical Examples

We use Project Data Sphere [82, 83] to download person‐level data for a randomized controlled trial with four trial arms.

### Practical Example A1

The VITamin D and OmegA‐3 TriaL (VITAL) was a large, randomized, factorial clinical trial involving 25 871 participants [84, 85, 86]. The primary goal of the study was to determine whether daily supplementation with vitamin D_3_ (2000 IU) or omega‐3 fatty acids (Omacor fish oil, 1 g) could reduce the risk of developing cancer, heart disease, or stroke among individuals with no prior history of these conditions. The trial had four trial arms (A: vitamin D placebo + fish oil placebo; B: vitamin D + fish oil placebo; C: vitamin D placebo + fish oil; D: vitamin D + fish oil). In this example, we calculated win proportions and win ratios using all‐cause death as an outcome. The results can be seen in the Table A1.

**TABLE A1:** Win Ratios and Win Proportions comparing time‐to‐death in randomized controlled trial NCT01169259, *N* = 25 871 with four treatment arms A (*N* = 6474), B (*N* = 6464), C (*N* = 6470) and D (*N* = 6463). ${\text{WP}}_{\mathrm{AD}}>\frac{1}{2},{\text{WP}}_{\mathrm{AB}}>\frac{1}{2},{\text{WP}}_{\mathrm{BC}}>\frac{1}{2}$, ${\text{WP}}_{\mathrm{DC}}>\frac{1}{2}$ and we have a transitive loop.

Compared arms	Win proportion (WP)	Win ratio (WR)
A vs. B, $T_{A}>T_{B}$	0.5001	1.0003
A vs. C, $T_{A}>T_{C}$	0.5027	1.0110
A vs. D, $T_{D}>T_{A}$	0.4943	0.9774
B vs. C, $T_{B}>T_{C}$	0.5026	1.0103
B vs. D, $T_{D}>T_{B}$	0.4941	0.9765
C vs. D, $T_{D}>T_{C}$	0.4914	0.9664

## Appendix K WR Approximation From Kaplan–Meier Curve

We digitized the Kaplan–Meier curve from BAATAF trial [48]. Then we used uniform inverse sampling to sample from the cdfs corresponding to the two treatment arms. We sampled 212 and 208 (matching the number of patients) and censored times beyond year 4. Afterwards, we estimated the WP and WR for each simulated dataset. Finally, we repeated the experiment 10 000 times and calculated the mean and median of the WR. In Case Study 1, the median is used. The code for our estimation can be found here: https://github.com/anna2313/KM.git.
